# Risk Factors for Septicemia Deaths and Disparities in a Longitudinal US Cohort

**DOI:** 10.1093/ofid/ofy305

**Published:** 2018-11-15

**Authors:** Jordan A Kempker, Michael R Kramer, Lance A Waller, Greg S Martin

**Affiliations:** 1 Division of Pulmonary, Allergy, Critical Care and Sleep Medicine, Emory University School of Medicine, Atlanta, Georgia; 2 Department of Epidemiology, Rollins School of Public Health, Emory University, Atlanta, Georgia; 3 Department of Biostatistics and Bioinformatics, Rollins School of Public Health, Emory University, Atlanta, Georgia

**Keywords:** epidemiology, sepsis, sepsis mortality

## Abstract

**Background:**

There are few longitudinal data on the risk factors and mediators of racial disparities in sepsis among community- dwelling US adults.

**Methods:**

This is a longitudinal study of adult participants in the 1999–2005 National Health Interview Survey with data linked to the 1999–2011 National Death Index. We utilized National Vital Statistics System’s ICD-10 schema to define septicemia deaths (A40-A41), utilizing influenza and pneumonia deaths (J09-J11) and other causes of death as descriptive comparators. All statistics utilized survey design variables to approximate the US adult population.

**Results:**

Of 206 691 adult survey participants, 1523 experienced a septicemia death. Factors associated with a >2-fold larger hazard of septicemia death included need for help with activities of daily living; self-reported “poor” and “fair” general health; lower education; lower poverty index ratio; self-reported emphysema, liver condition, stroke, and weak or failing kidneys; numerous measures of disability; general health worse than the year prior; >1 pack per day cigarette use; and higher utilization of health care. Blacks had age- and sex-adjusted hazards that were higher for septicemia deaths (hazard ratio [HR], 1.92; 95% confidence interval [CI], 1.65–2.23) than for other causes of death (HR, 1.32; 95% CI, 1.25–1.38). The strongest mediators of the septicemia disparity included self-reported general health condition, family income-poverty ratio, and highest education level achieved.

**Conclusions:**

In this cohort, the major risk factors for septicemia death were similar to those for other causes of death, there was approximately a 2-fold black-white disparity in septicemia deaths, and the strongest mediators of this disparity were across domains of socioeconomic status.

Sepsis is defined as a dysregulated immune response to infection that results in acute organ dysfunction [1]. Amelioration measures to date have focused on treatment of patients during the acute phase of illness, prevention of hospital-acquired infections, and the lasting sequelae of sepsis survivors; however, there is a need to further understand the extent to which community-acquired sepsis is a preventable condition. This is in line with public health agencies’ recent increased attention to the burden of sepsis. Specifically, in 2017, the World Health Organization officially recognized sepsis as a major global health problem, and the Centers for Disease Control and Prevention launched a US initiative aimed at reducing the burden of sepsis [2, 3]. In the United States, in addition to understanding the risk factors associated with the high morbidity of disease, there is also a need to understand the significant social disparities. Specifically, observations from administrative data have demonstrated that blacks, when compared with whites, suffer a 1.5- to 3.5-fold increase in the risk of sepsis incidence and sepsis-related mortality [4–13]. A notable exception to these findings is a cohort study of emergency room encounters in a large US population–based cohort, which demonstrated that blacks were less likely than whites to suffer sepsis events [14]. Given the methodological strength of this outlier, the associations between race/ethnicity and sepsis require continued study in longitudinal cohorts.

Descriptive analyses are needed as first steps to explore and understand the potential population-level risk factors for sepsis and social disparities in sepsis among community-dwelling individuals. The goals of this study are to use a large cohort of community-dwelling US adults to (1) broadly explore and describe the health conditions, health behaviors, and socioeconomic factors associated with increased risk for subsequent sepsis mortality and (2) describe which factors are associated with the race/ethnicity disparities in sepsis death. We approach these objectives from a biopsychosocial framework of human health, organizing potential risk factors in a complex web of biological and social determinants of health [15]. Although social determinants in overall mortality have been extensively studied, fundamental social causes of disease theory suggest that social determinants of health continue to be flexibly and differentially reproduced in different disease processes within specific contexts, arguing for ongoing exploration of this theme in specific disease states [16]. In regards to sepsis, prior studies have relied primarily on cross-sectional designs, and they had insufficient individual-level data or longitudinal data on factors to be able to explore which may play important roles in the risk of sepsis mortality [17].

## METHODS

We examined data from a longitudinal cohort of adult participants in the 1999–2005 National Health Interview Survey (NHIS) with sufficient identifier data to permit linkage to records in the 1999–2011 National Death Index (NDI).

### Setting and Participants

The NHIS is an annually evolving National Center for Health Statistics (NCHS) survey that deploys a decennially updated multistage area probability sampling strategy. Additionally, the NHIS oversamples minority groups to improve precision and accuracy of national statistical estimates of the entire civilian, noninstitutionalized population. For this study, we used data from participants of the Sample Adult NHIS, which is administered to 1 randomly selected individual ≥18 years old per each selected household [18]. Mortality outcomes for this cohort are obtained through the NDI, the NCHS’s centralized system of death certificate information. The NCHS uses 14 identifiers to perform the data linkage before de-identifying the data for research use [19]. We excluded NHIS participants who did not have sufficient baseline survey data to be eligible for the NDI linkage procedures. For the remaining participants eligible for linkage, the NCHS creates new eligibility-adjusted weights. We further excluded those who were linked but with missing date or cause of death information.

The general analytic approach was descriptive, estimating associations between baseline survey characteristics and the subsequent risk of cause-specific death. These associations were examined separately for septicemia death, influenza/pneumonia death, and aggregated other causes of death. This method allows comparisons of the specificity of associations across the 2 major ICD-10 groupings that conceptually encompass the majority of sepsis in the United States with a grouping that largely encompasses non–infectious diseases–related death. Additionally, we elected to analyze septicemia and influenza/pneumonia deaths separately to allow this analysis to be congruent with the National Vital Statistics System’s code groupings and annual mortality reports for these conditions. Specifically, we used the septicemia code grouping (A40-A41) to define a septicemia death as a death with any of these codes listed among any of the causes of death on the death record [20]. An influenza/pneumonia death was similarly defined using the J09-J11 ICD-10 codes. Any death that did not meet either the septicemia or influenza/pneumonia definitions was categorized as an “other death.” Eligible participants without a linked death record were assumed to be alive and censored on December 31, 2011. Time to death was calculated from the date of the baseline survey. To organize the variables, we group them into domains of health condition, medical comorbidity, health behaviors, and socioeconomic status (SES). SES is further subdivided into domains of material capital, social capital, and human capital [15]. For race/ethnicity, we utilized the 4-category NHIS variable that categorizes participants as “Hispanic,” “Non-Hispanic White,” “Non-Hispanic Black,” or “Non-Hispanic; All other race groups.” The remainder of this manuscript refers to non-Hispanic black and non-Hispanic white as black and white, respectively.

### Statistical Methods

This study was approved by the Research Data Center of the NCHS (proposal number 1321). Data were accessed and analyzed by J.A.K. on-site at the secure computer laboratories of the Census Bureau’s Atlanta Research Data Center, and statistical output was reviewed by an NCHS analyst before release. Due to NCHS data policies, unweighted sample sizes are reported only for the entire cohort and largest, relevant subdivisions, whereas the rest of the report is in weighted, aggregate statistics. All analyses were performed using the SAS 9.2 (SAS Institute, Cary, NC) survey procedures accounting for design variables and eligibility-adjusted sample weights. These weights, intended for annual estimates, were divided by 7 to account for the 7 years of data. Graphs were created with *ggplot2* (v2.2.1) and *forestplot* (v1.7) in RStudio 0.99.90 (RStudio Inc., Boston, MA).

For descriptive analyses, we started with a Cox proportional hazards model for time to outcome that included age group and sex. This baseline model was run for 3 separate outcomes: (1) a model for septicemia deaths, with influenza/pneumonia and other deaths censored; (2) a model for influenza/pneumonia deaths, with septicemia and other deaths censored; and (3) a model for other deaths, with both septicemia and influenza/pneumonia deaths censored. Next, we added in each baseline characteristic, 1 at a time, into these 3 age- and sex-adjusted models. The output is presented as age- and sex-adjusted hazard ratios (HRs) with accompanying 95% confidence intervals (CIs).

**Figure 1.**
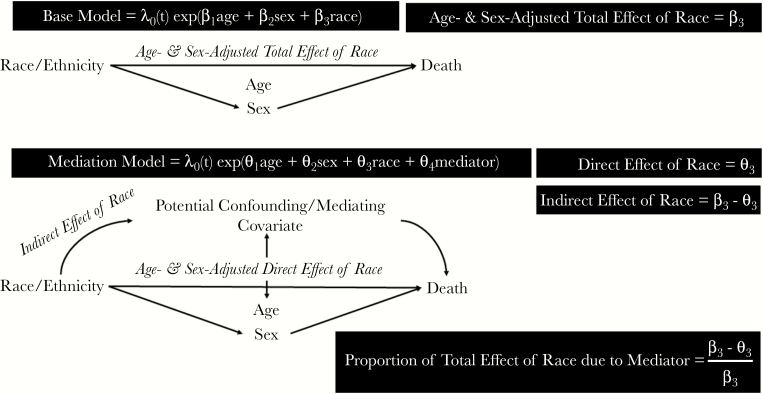
Framework for mediation analyses.

For the mediation analyses, we decomposed the total age- and sex-adjusted race/ethnicity parameter estimate, interpreted as the total magnitude of the observed racial inequality adjusting for age and sex distribution. When each covariate was added to the age-, sex-, and race/ethnicity-adjusted model, the new race/ethnicity parameter estimate was then taken as the direct effect and conceptualized as the residual race/ethnicity inequality not explained by the added covariate. Subtracting this direct effect from the total effect produced the indirect effect, conceptualized as the amount of the total effect explained by the added covariate [21]. In addition to this absolute measure, we calculated the proportion of the total effect explained by the indirect effect of a covariate (Figure 1). We focused the mediation analyses on the outcomes of septicemia and other causes of death and the race/ethnicities of black and white. The decision to focus on the black-white disparity was made a priori given the conflicting data in the existing sepsis literature, noted above. The decision to exclude influenza/pneumonia death from the mediation analyses was due to the fact that initial analyses revealed that the 95% CI of the hazard ratio for influenza/pneumonia death in blacks compared with whites already included the null effect.

## RESULTS

There were 221 151 participants in the 1999–2005 Sample Adult NHIS survey, with 206 691 participants included in the analyses. The primary reason for exclusion was lack of sufficient NHIS identifiers for NDI linkage. To avoid the potential for unequal probabilities of missing linkage information, the NCHS reweights the remaining study sample to approximate the US population. From this resultant sample, 82 participants were additionally excluded, without reweighting, for missing information about date and cause of death. By the end of the 2011, 183 236 (89%) individuals from the cohort were still alive with a mean of 9.4 years of follow-up, 1523 (1%) experienced a septicemia death with a mean of 5.1 years of follow-up, 1785 (1%) experienced an influenza/pneumonia death with a mean of 5.1 years of follow-up, and 20 450 (10%) experienced a death from another cause with a mean of 5.1 years of follow-up (Supplementary Figure 1).

### Age Group– and Sex-Adjusted Hazards by Cause of Death

**Figure 2.**
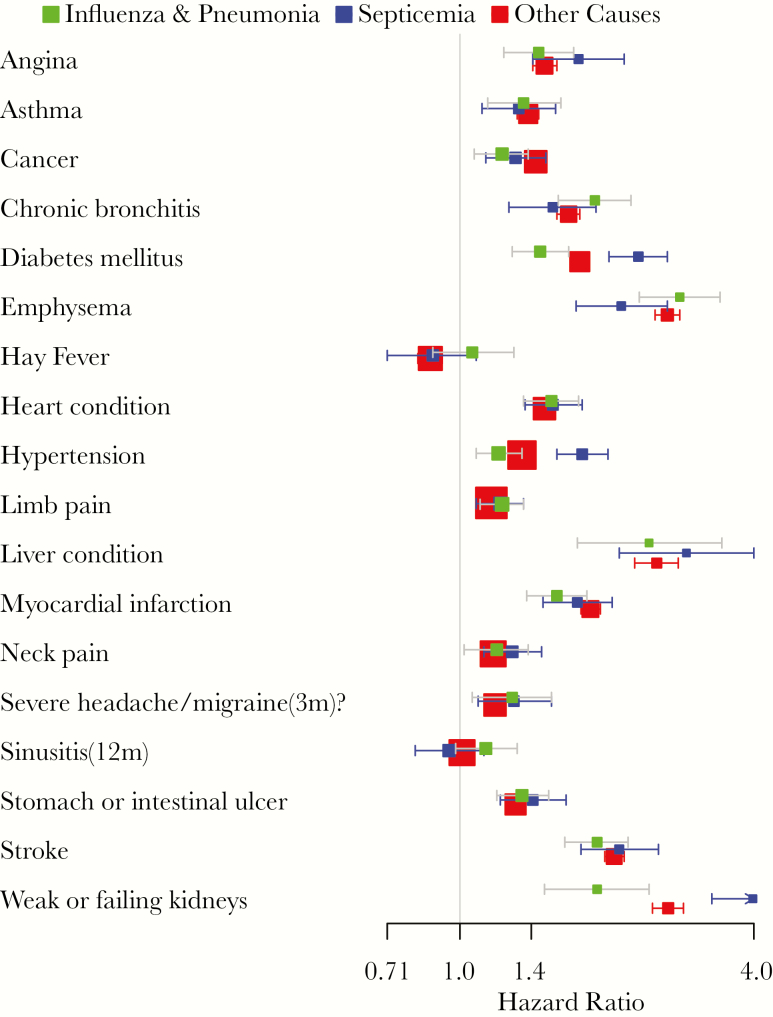
Age group– and sex-adjusted medical comorbidity hazards for death, National Health Interview Survey 1999–2005. Graph includes results of 3 separate Cox proportional hazards models in the same cohort: 1 for septicemia (A40-A41) deaths that censors for other causes of death (blue), 1 for influenza/pneumonia (J09-J11) deaths that censors for other causes of death (red), and 1 for other causes of death that censors for septicemia and influenza/pneumonia deaths (green). Whiskers denote 95% confidence intervals.

The age- and sex-adjusted analyses are presented in full in the Supplementary Data (see Supplementary Tables 1–3, Supplementary Figures 2–9, and (in space-limited form) Table 1). The factors associated with larger age- and sex-adjusted hazards of death were similar across all 3 death outcomes. Factors that were associated with an approximately 4-fold or larger hazard of death across all 3 groupings included self-reported need for help with activities of daily living and self- reported “poor” general health. Factors that were associated with an approximately 2–4-fold greater hazard of death across all 3 groupings included lower level of education achieved; lower poverty index ratio (annual family income divided by national poverty level for family size); self-reported emphysema, liver condition, stroke, and weak or failing kidneys (Figure 2); numerous self-reported measures of disability; self-reported “fair” general health and general health worse than the year prior; >1 pack per day cigarette use; and higher utilization of health care encounters. Factors that were associated with an approximately 2-fold lower hazard of death across all 3 groupings only included self-reported weekly vigorous exercise.

**Table 1.** Baseline Survey Characteristics: Age Group– and Sex-Adjusted Hazards for Cause-Specific Death, National Health Interview Survey 1999–2005^a^

Variable/Response	Influenza and Pneumonia HR (95% CI)	Septicemia HR (95% CI)	Other HR (95% CI)
Human capital			
Race/ethnicity			
Hispanic	0.95 (0.77–1.16)	**1.35 (1.13–1.62**)	**0.82 (0.78–0.86**)
Non-Hispanic, all other race groups	0.8 (0.55–1.16)	1.16 (0.82–1.66)	**0.82 (0.74–0.92**)
Non-Hispanic black	1.14 (0.96–1.36)	**1.92 (1.65–2.23**)	**1.32 (1.25–1.38**)
Non-Hispanic white	Ref	Ref	Ref
Not born in the United States	**0.79 (0.67–0.95**)	0.86 (0.72–1.03)	**0.72 (0.69–0.76**)
Not a citizen of the United States	0.92 (0.68–1.24)	1.07 (0.79–1.45)	**0.75 (0.68–0.82**)
Language of the interview			
English	Ref	Ref	Ref
English and Spanish	0.81 (0.51–1.31)	1.24 (0.8–1.91)	**0.76 (0.66–0.86**)
Spanish	0.98 (0.72–1.34)	1.2 (0.89–1.62)	**0.81 (0.75–0.88**)
Education			
Never attended/kindergarten only	1.8 (0.75–4.33)	**2.89 (1.19–7.01**)	**2.02 (1.56–2.61**)
Grades 1–11	**3.07 (1.62–5.82**)	**2.53 (1.22–5.23**)	**2.57 (2.11–3.12**)
12th grade, no diploma	1.46 (0.69–3.1)	**2.52 (1.15–5.57**)	**2.22 (1.79–2.76**)
High school graduate	**2.09 (1.09–4.01**)	1.78 (0.86–3.67)	**1.93 (1.58–2.35**)
GED or equivalent	**1.97 (1.01–3.82**)	1.86 (0.87–3.95)	**1.95 (1.59–2.38**)
Some college, no degree	**2.16 (1.13–4.12**)	1.44 (0.69–3.03)	**1.81 (1.48–2.21**)
AA degree: technical or vocational	1.75 (0.88–3.48)	1.38 (0.63–3)	**1.82 (1.48–2.25**)
AA degree: academic program	1.39 (0.59–3.27)	1.1 (0.46–2.63)	**1.62 (1.29–2.03**)
Bachelor’s degree (BA, AB, BS, BBA)	1.53 (0.79–2.98)	1.18 (0.55–2.53)	**1.26 (1.03–1.55**)
Master’s degree (MA, MS, MEng, MEd, MBA)	1.04 (0.51–2.11)	0.96 (0.45–2.02)	1.18 (0.96–1.45)
Professional degree (MD, DDS, DVM, JD)	1.33 (0.59–2.98)	1.08 (0.4–2.92)	1.16 (0.92–1.47)
Doctoral degree (PhD, EdD)	Ref	Ref	Ref
Social capital			
Living with a significant other	**0.61 (0.54–0.68**)	**0.62 (0.55–0.7**)	**0.65 (0.63–0.67**)
Family type			
Multiple adults, ≥1 child	**0.64 (0.5–0.84**)	**0.6 (0.47–0.77**)	**0.63 (0.59–0.67**)
Multiple adults	**0.73 (0.65–0.81**)	**0.78 (0.69–0.88**)	**0.73 (0.7–0.76**)
One adult, ≥1 child	1.39 (0.91–2.13)	0.82 (0.53–1.27)	**0.81 (0.71–0.91**)
One adult	Ref	Ref	Ref
Material capital			
Poverty index ratio			
<0.50	**3 (2.1–4.3**)	**3.37 (2.41–4.73**)	**2.38 (2.14–2.64**)
0.50–0.74	**3.63 (2.64–4.98**)	**3.9 (2.98–5.11**)	**2.92 (2.67–3.19**)
0.75–0.99	**3.76 (2.86–4.94**)	**4.12 (3.21–5.28**)	**2.58 (2.38–2.78**)
1.00–1.24	**3.29 (2.52–4.3**)	**2.7 (2.05–3.55**)	**2.45 (2.26–2.65**)
1.25–1.49	**3.03 (2.35–3.91**)	**2.09 (1.54–2.83**)	**2.32 (2.15–2.51**)
1.50–1.74	**2.14 (1.63–2.81**)	**2.12 (1.55–2.91**)	**1.98 (1.82–2.15**)
1.75–1.99	**1.83 (1.33–2.51**)	**2.08 (1.53–2.84**)	**1.81 (1.66–1.98**)
2.00–2.49	**2.21 (1.74–2.81**)	**1.62 (1.24–2.11**)	**1.8 (1.68–1.93**)
2.50–2.99	**1.73 (1.29–2.33**)	**1.96 (1.48–2.6**)	**1.65 (1.52–1.78**)
3.00–3.49	**1.96 (1.45–2.66**)	**1.53 (1.11–2.12**)	**1.59 (1.46–1.73**)
3.50–3.99	**1.49 (1.07–2.07**)	1.35 (0.95–1.92)	**1.38 (1.26–1.5**)
4.00–4.49	1.33 (0.88–2.02)	1.29 (0.86–1.95)	**1.3 (1.18–1.44**)
4.50–4.99	1.02 (0.64–1.61)	0.79 (0.5–1.26)	**1.15 (1.03–1.29**)
≥5.00	Ref	Ref	Ref
Home ownership			
Other arrangement	**1.88 (1.46–2.41**)	1.37 (0.99–1.9)	**1.61 (1.48–1.75**)
Rented	**1.49 (1.31–1.68**)	**1.7 (1.5–1.91**)	**1.53 (1.47–1.58**)
Owned or being bought	Ref	Ref	Ref
No telephone number in house	**1.49 (1.01–2.19**)	**1.65 (1.18–2.3**)	**2.02 (1.84–2.21**)
No health insurance any time, past year	0.71 (0.38–1.31)	1.19 (0.76–1.89)	**1.27 (1.13–1.43**)
Delayed care in past year because of worry of cost	**1.49 (1.21–1.82**)	**1.38 (1.15–1.65**)	**1.36 (1.28–1.44**)
Foregone health care in past year because of cost	**1.77 (1.42–2.19**)	**1.71 (1.36–2.14**)	**1.6 (1.5–1.7**)
Health condition			
Medical history			
Angina, ever	**1.45 (1.23–1.71**)	**1.75 (1.41–2.17**)	**1.49 (1.41–1.58**)
Asthma, ever	**1.35 (1.14–1.61**)	**1.32 (1.11–1.57**)	**1.38 (1.31–1.45**)
Cancer, past year	**1.22 (1.07–1.38**)	**1.3 (1.13–1.5**)	**1.43 (1.38–1.49**)
Chronic bronchitis, past year	**1.89 (1.59–2.24**)	**1.55 (1.26–1.9**)	**1.67 (1.58–1.76**)
Diabetes mellitus, past year	**1.46 (1.28–1.67**)	**2.32 (2.02–2.66**)	**1.76 (1.69–1.82**)
Emphysema, ever	**2.82 (2.33–3.41**)	**2.14 (1.73–2.66**)	**2.66 (2.51–2.82**)
Hay fever, past year	1.06 (0.88–1.29)	0.88 (0.71–1.08)	0.87 (0.82–0.92)
Heart condition, ever	**1.54 (1.35–1.75**)	**1.55 (1.36–1.78**)	**1.49 (1.43–1.54**)
Hypertension, ever	**1.2 (1.08–1.34**)	**1.78 (1.58–2.01**)	**1.34 (1.3–1.38**)
Limb pain	**1.22 (1.1–1.35**)	**1.21 (1.08–1.35**)	**1.16 (1.12–1.19**)
Liver condition, past year	**2.44 (1.74–3.44**)	**2.91 (2.12–4**)	**2.53 (2.28–2.8**)
Myocardial infarction, ever	**1.58 (1.37–1.82**)	**1.74 (1.48–2.05**)	**1.85 (1.77–1.94**)
Neck pain, 3 mo	**1.19 (1.02–1.38**)	**1.28 (1.12–1.47**)	**1.17 (1.13–1.22**)
Severe headaches/migraines, 3 mo	**1.28 (1.06–1.54**)	**1.29 (1.09–1.54**)	**1.18 (1.13–1.24**)
Sinusitis, past year	1.13 (0.98–1.31)	0.95 (0.81–1.12)	1.01 (0.97–1.06)
Ulcer, ever	**1.34 (1.19–1.52**)	**1.41 (1.21–1.65**)	**1.3 (1.24–1.36**)
Stroke, ever	**1.91 (1.64–2.21**)	**2.12 (1.77–2.55**)	**2.07 (1.98–2.17**)
Weak or failing kidneys	**1.91 (1.49–2.44**)	**3.98 (3.28–4.82**)	**2.67 (2.48–2.87**)
Body mass index, kg/m^2^			
<18.5	**1.7 (1.43–2.02**)	**1.71 (1.4–2.1**)	**1.36 (1.28–1.46**)
18.5–24.9	Ref	Ref	Ref
25–29.9	**0.66 (0.59–0.75**)	**0.86 (0.75–0.99**)	**0.8 (0.77–0.82**)
30–34.9	**0.73 (0.62–0.86**)	1.14 (0.97–1.34)	**0.86 (0.83–0.9**)
≥35	1.1 (0.9–1.34)	**1.8 (1.46–2.22**)	**1.25 (1.18–1.33**)
Any limitation, all conditions	**2.87 (2.55–3.23**)	**3.2 (2.85–3.6**)	**2.59 (2.51–2.66**)
Any functional limitation, all conditions	**2.16 (1.9–2.46**)	**2.28 (2.01–2.58**)	**1.97 (1.9–2.04**)
Need help with ADLs	**4.34 (3.74–5.03**)	**4.75 (4.02–5.61**)	**3.37 (3.16–3.59**)
Need help with instrumental ADLs	**3.57 (3.14–4.06**)	**3.19 (2.78–3.66**)	**2.88 (2.75–3.01**)
Health problem requires special equipment	**2.72 (2.39–3.09**)	**3.1 (2.72–3.52**)	**2.5 (2.41–2.6**)
Unable to work due to health problem	**2.82 (2.5–3.18**)	**3.16 (2.8–3.58**)	**2.65 (2.56–2.75**)
Self-reported general health			
Poor	**5.18 (4.11–6.55**)	**7.93 (6.15–10.22**)	**5.31 (4.98–5.67**)
Fair	**3 (2.43–3.71**)	**4.73 (3.78–5.93**)	**2.95 (2.79–3.11**)
Good	**1.8 (1.47–2.2**)	**2.75 (2.21–3.41**)	**1.87 (1.78–1.97**)
Very good	1.22 (0.98–1.51)	**1.5 (1.18–1.91**)	**1.34 (1.27–1.41**)
Excellent	Ref	Ref	Ref
Health compared with a year ago			
Better	1.12 (0.96–1.31)	**1.3 (1.12–1.52**)	**1.19 (1.13–1.24**)
About the same	Ref	Ref	Ref
Worse	**2 (1.75–2.29**)	**1.93 (1.68–2.21**)	**1.98 (1.9–2.06**)
Days health kept patient in bed, past year			
None	Ref	Ref	Ref
1–7	1.12 (0.96–1.3)	1.04 (0.88–1.22)	**1.09 (1.05–1.14**)
>7	**2.19 (1.9–2.52**)	**2.44 (2.12–2.82**)	**2.16 (2.08–2.25**)
Health behaviors			
Cigarettes packs per day, all participants			
None	Ref	Ref	Ref
0.5	**1.7 (1.4–2.05**)	**1.57 (1.27–1.93**)	**1.83 (1.74–1.93**)
0.5–1	**1.64 (1.34–2**)	**1.71 (1.42–2.06**)	**2.12 (2.01–2.23**)
>1	**2.46 (1.85–3.28**)	**2.36 (1.83–3.04**)	**2.85 (2.65–3.06**)
Alcohol use, d/wk^b^			
None	Ref	Ref	Ref
1–2	**0.62 (0.52–0.75**)	**0.65 (0.53–0.79**)	**0.74 (0.71–0.78**)
3–5	**0.58 (0.44–0.77**)	**0.48 (0.35–0.66**)	**0.73 (0.68–0.78**)
6–7	**0.69 (0.56–0.85**)	**0.77 (0.6–0.98**)	**0.92 (0.87–0.98**)
Light or moderate activity, times/wk			
None	Ref	Ref	Ref
1–3	**0.59 (0.5–0.69**)	**0.54 (0.46–0.63**)	**0.58 (0.56–0.6**)
4–7	**0.59 (0.52–0.67**)	**0.55 (0.46–0.65**)	**0.6 (0.58–0.62**)
>7	**0.7 (0.49–1**)	**0.58 (0.4–0.85**)	**0.74 (0.66–0.82**)
Muscle strengthening activity, times/wk			
None	Ref	Ref	Ref
1–3	**0.48 (0.36–0.62**)	**0.62 (0.48–0.8**)	**0.59 (0.55–0.63**)
4–7	0.8 (0.62–1.04)	**0.69 (0.53–0.88**)	**0.68 (0.63–0.73**)
>7	0.78 (0.36–1.66)	0.93 (0.47–1.86)	0.95 (0.76–1.17)
Vigorous activity, times/wk			
None	Ref	Ref	Ref
1–3	**0.38 (0.3–0.48**)	**0.42 (0.34–0.52**)	**0.51 (0.48–0.54**)
4–7	**0.38 (0.29–0.51**)	**0.35 (0.26–0.47**)	**0.5 (0.46–0.53**)
>7	**0.3 (0.11–0.8**)	0.84 (0.37–1.92)	**0.61 (0.48–0.77**)
Health care utilization			
Has usual place for health care	1 (0.77–1.31)	1.13 (0.89–1.42)	0.97 (0.92–1.03)
No. of office visits, past year			
None	Ref	Ref	Ref
1	0.76 (0.58–1)	0.92 (0.7–1.2)	**0.86 (0.81–0.92**)
2–3	1 (0.81–1.24)	0.83 (0.67–1.02)	**0.87 (0.82–0.92**)
>3	**1.35 (1.11–1.65**)	**1.62 (1.34–1.96**)	**1.36 (1.29–1.44**)
Received health care >10 times, past year	**1.87 (1.68–2.08**)	**2.4 (2.15–2.68**)	**1.89 (1.83–1.95**)
Overnight hospital stays, past year			
None	Ref	Ref	Ref
1	**1.82 (1.57–2.1**)	**1.91 (1.64–2.22**)	**1.66 (1.59–1.73**)
>1	**2.43 (2.01–2.94**)	**3.4 (2.83–4.08**)	**2.87 (2.71–3.03**)
No. of ER visits, past year			
None	Ref	Ref	Ref
1	**1.47 (1.28–1.68**)	**1.54 (1.32–1.79**)	**1.45 (1.39–1.52**)
2–3	**2.14 (1.8–2.54**)	**2.26 (1.86–2.75**)	**2 (1.89–2.12**)
>3	**2.42 (1.79–3.28**)	**3.56 (2.79–4.55**)	**2.88 (2.64–3.13**)
Received pneumonia vaccine in lifetime	**1.14 (1.02–1.28**)	**1.17 (1.01–1.34**)	**1.18 (1.14–1.23**)
Received influenza vaccine, past year	**1.14 (1.01–1.28**)	1.11 (0.98–1.26)	**1.07 (1.03–1.1**)

Abbreviations: AA, Associates in Arts; ADL, activity of daily living; CI, confidence interval; ER, emergency room; HR, hazard ratio; GED, General Educational Development.

^a^The cells that are in bold include hazard ratios with 95% confidence intervals that do not include 1.0.

^b^Lifetime abstainers and those who had not drunk in the past year recoded as 0.

In regards to race/ethnicity, when compared with whites, Hispanics had age- and sex-adjusted hazards that were lower for other causes of death (HR, 0.82; 95% CI, 0.78–0.86), similar for influenza/pneumonia deaths (HR, 0.95; 95% CI, 0.77–1.16), and larger for septicemia deaths (HR, 1.35; 95% CI, 1.13–1.62). Blacks had age- and sex-adjusted hazards that were higher for other causes of death (HR, 1.32; 95% CI, 1.25–1.38), similar for influenza/pneumonia deaths (HR, 1.14; 95% CI, 0.96–1.36), and higher for septicemia deaths (HR, 1.92; 95% CI, 1.65–2.23). This black-white disparity was further explored in 2 stratified analyses: an age-adjusted, sex-stratified model and a sex-adjusted, age-stratified model. These models did not demonstrate appreciable interaction between the black-white disparity when stratified by age group or sex (Supplementary Tables 4 and 5).

### Mediation Analyses for Black-White Hazards for Septicemia and Other Deaths

**Figure 3.**
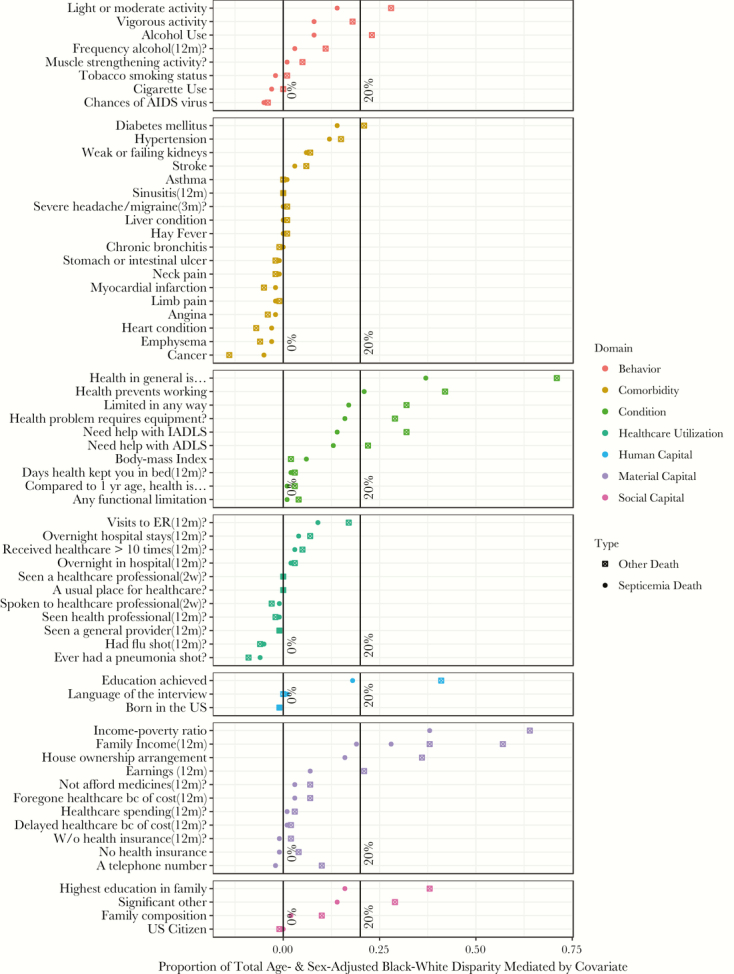
Comparisons of covariates’ proportional mediation of the black-white disparities for septicemia and other causes of death, National Health Interview Survey 1999–2005. The proportions for this graph are calculated by dividing the indirect effect of the age- and sex-adjusted black-white disparity when adjusting for a particular mediator by the total age- and sex-adjusted black-white disparity without adjustment for that mediator. This is conceptualized as the relative amount of the total age- and sex-adjusted black-white disparity accounted for by the specific variable. This is performed separately for septicemia and other causes of death to calculate cause of death–specific proportional mediation for each variable. Abbreviations: ADL, activity of daily living; ER, emergency room; IADL, instrumental activity of daily living.

The factors that explained the age- and sex-adjusted racial disparity in blacks compared with whites were similar for septicemia and other causes of death (Figure 3). In general, a larger absolute amount (indirect effect) of the black-white disparity was explained by covariate adjustments for septicemia deaths than for other causes of death. Similarly, the residual black-white disparity (direct effects) after covariate adjustments was also generally larger for septicemia deaths than for other causes of death (Supplementary Table 6, Supplementary Figure 10). In contrast, the relative proportion of the black-white disparity explained by covariate adjustments was generally smaller for septicemia deaths than for other causes of death.

The most pronounced mediators of black-white disparities for both septicemia and other causes of death were self- reported general health condition, self-reported disability, and several factors across every domain of socioeconomic status. Notably, for the black-white disparities in septicemia and other causes of death, respectively, self-reported general health condition accounted for 37% and 71% of the disparity, family income-poverty ratio accounted for 38% and 64% of the disparity, and highest education level achieved accounted for 41% and 18% of the disparity (Supplementary Table 6).

## DISCUSSION

To our knowledge, this analysis is the first to use a large, nationally representative longitudinal cohort of community-dwelling adults to broadly describe the baseline risk factors that (1) are associated with subsequent hazards for septicemia death and (2) mediate the black-white disparity in septicemia death. First, we observe that the major risk factors for septicemia death are similar to those for other causes of death: poor general health condition; low socioeconomic status (SES); disability; chronic diseases such as emphysema, stroke, and kidney and liver disease; heavy cigarette use; and higher frequency of health care encounters. Second, there was approximately a 2-fold black-white disparity in septicemia deaths—larger than the disparity for other causes of death and in contrast to the absence of a demonstrated disparity in influenza/pneumonia deaths. Finally, the strongest mediators of these disparities were across domains of SES.

This study has several strengths. It benefits from the robust sampling and survey procedures of the NCHS in producing a large, multiyear cohort of US community-dwelling adults that is highly generalizable to this population and has sufficient follow-up time to have an adequate number of events for analyses. The follow-up is adequate, with 93% of the eligible sample having sufficient data for accurate linkage with the nation’s most comprehensive database of death records. The exploratory objective of the study benefits from the extensive questionnaires of the NCHS and thus is able to evaluate a broad number of potential risk factors across domains of health conditions, health behaviors, health care utilization, and many components of SES.

Before speculating on the interpretation of the findings, we place them in the context of the study’s limitations. First, exposures are self-reported and therefore may suffer from imprecise or incorrect values. Second, septicemia death is a conditional outcome in that it is indicative of the entire sequence from acquisition of infection, the development of septicemia, and death. It is not possible in this data set to distinguish associations specifically attributable to each step in this sequence. Third, this large cohort likely underestimates or does not include rare risk factors that are not represented in an adequate frequency among the approximately 200 000 community-dwelling participants willing and able to participate in the survey. For example, severe immunodeficiencies are a conceivable risk factor for septicemia but are not represented adequately in this cohort due to low prevalence.

Another limitation is the lack of a validated ICD-10 definition for sepsis death in the NDI. Instead of introducing a definition our data set was not equipped to validate, we utilized the longstanding National Vital Statistics System ICD-10 septicemia definition and its influenza/pneumonia definition as a comparator. These definitions are likely sensitive but not specific to the full spectrum of clinically defined sepsis. With national and global health agencies now addressing sepsis as a public health problem, we feel that using these case definitions allows comparisons with US national mortality statistics dating back to 1999, which is more helpful in developing consistent public health policy in this area. Sensitivity analyses combining septicemia and influenza/pneumonia deaths into a single sepsis definition demonstrate similar results with predictably narrower confidence intervals (results available on request).

Our novel findings are important for several reasons. First, at a population level, the factors associated with increased risk of septicemia deaths are similar to those associated with risk for other causes of death. The most prominent factors are familiar: low SES measures such as education and poverty, chronic illnesses marked by major organ dysfunction, functional disability, tobacco use, and higher frequencies of health care encounters. The strength of association of an individual’s own perception of general health with subsequent mortality has been demonstrated before, and our speculation is that this reflects a composite of one’s health, function, and SES [22]. Finally, whether factors such as diabetes mellitus, kidney disease, hypertension, and morbid obesity convey excess risk specific to septicemia death in comparison with influenza/pneumonia and other deaths (by the greater magnitude of their septicemia hazard ratios with nonoverlapping CIs) are provocative and hypothesis-generating findings.

The observations that blacks suffer higher age- and sex-adjusted mortality than whites are consistent with the National Vital Statistics’ 2015 finding that “[t]he age-adjusted death rate has been 1.2 times greater for the non-Hispanic black population than for the non-Hispanic white population since 2008” [23]. Our finding that this disparity is larger for septicemia deaths than for other causes of death (age- and sex- adjusted HRs [95% CIs] 1.92 [1.65–2.23] and 1.32 [1.25–1.38] times higher, respectively) is also consistent with the NVSS’s 2015 demonstration of septicemia age-adjusted death rates of 18.6 and 10.1 per 100 000 person-years among blacks and whites, respectively [23]. Although this larger septicemia disparity is consistent with the majority of the published literature, it appears to be in contrast with 1 large, US prospective cohort study of sepsis incidence by Moore et al. [4–14]. The different results may be due to differences in design. The Moore et al. study is a cohort of >45-year-olds that assesses the incidence of community-acquired sepsis in emergency departments, whereas our study cohort is >18 years old, includes both community- and health care–acquired sepsis, and assesses sepsis deaths. This last point again highlights that our study outcome captures the entire chain of events from acquisition of infection to development of sepsis acute organ dysfunction to death. Therefore, the disparity may occur at any of the steps along this pathway, and there are insufficient data to completely untangle this web of causation. Our present study’s contribution to this literature is the confirmation of an elevated black-white septicemia mortality disparity and additional evidence regarding the roles the major moderators of this disparity lying within a complex web of disparities in SES. Although these data were not equipped to assess the role of potential genetic differences in the risk for sepsis deaths, the diminished age- and sex-adjusted black-white disparity when adjusting for SES suggests that at a population level these genetic factors do not play a major role in this disparity. Finally, these data demonstrate that Hispanics had higher hazards of septicemia death in comparison with non-Hispanic whites but lower hazards of other causes of death and no differences in hazards of influenza/pneumonia death—a provocative hypothesis-generating observation.

In conclusion, this study demonstrates that in the United States (1) the elderly, frail, and impoverished suffer the highest excess risk of septicemia and influenza/pneumonia deaths when compared with deaths from other causes; (2) blacks suffer a disproportionately elevated septicemia mortality risk in comparison with other aggregated causes of death and influenza/pneumonia death; and (3) the largest moderators of this racial disparity are within the domains of SES. These findings support the longstanding notion that social status and health are interwoven threads of an intricate web of causation influencing health outcomes within a population. They support the concept that the goal of equitable health outcomes will be achieved only through a comprehensive public health strategy that incorporates and strives to correct social inequalities.

## Supplementary Data

Supplementary materials are available at *Open Forum Infectious Diseases* online. Consisting of data provided by the authors to benefit the reader, the posted materials are not copyedited and are the sole responsibility of the authors, so questions or comments should be addressed to the corresponding author.

ofy305_suppl_supplementary_material

## References

[1] Singer M, Deutschman CS, Seymour CW, et al The third international consensus definitions for sepsis and septic shock (Sepsis-3). JAMA 2016; 315:801–10. 26903338 10.1001/jama.2016.0287 PMC4968574

[2] The World Health Organization. Improving the prevention, diagnosis and management of sepsis http://apps.who.int/gb/ebwha/pdf_files/EB140/B140_R5-en.pdf. Accessed 10 August 2017.

[3] Centers for Disease Control and Prevention. CDC urges early recognition, prompt treatment of sepsis 2017 https://www.cdc.gov/media/releases/2017/p0831-sepsis-recognition-treatment.html. Accessed 29 Novermber 2017.

[4] Barnato AE, Alexander SL, Linde-Zwirble WT, Angus DC Racial variation in the incidence, care, and outcomes of severe sepsis: analysis of population, patient, and hospital characteristics. Am J Respir Crit Care Med 2008; 177:279–84. 17975201 10.1164/rccm.200703-480OC PMC2720103

[5] Dombrovskiy VY, Martin AA, Sunderram J, Paz HL Occurrence and outcomes of sepsis: influence of race. Crit Care Med 2007; 35:763–8. 17255870 10.1097/01.CCM.0000256726.80998.BF

[6] Martin GS, Mannino DM, Eaton S, Moss M The epidemiology of sepsis in the United States from 1979 through 2000. N Engl J Med 2003; 348:1546–54. 12700374 10.1056/NEJMoa022139

[7] Melamed A, Sorvillo FJ The burden of sepsis-associated mortality in the United States from 1999 to 2005: an analysis of multiple-cause-of-death data. Crit Care 2009; 13:R28. 19250547 10.1186/cc7733 PMC2688146

[8] Baine WB, Yu W, Summe JP The epidemiology of hospitalization of elderly Americans for septicemia or bacteremia in 1991-1998. Application of Medicare claims data. Ann Epidemiol 2001; 11:118–26. 11164128 10.1016/s1047-2797(00)00184-8

[9] Burton DC, Flannery B, Bennett NM, et al; Active Bacterial Core Surveillance/Emerging Infections Program Network Socioeconomic and racial/ethnic disparities in the incidence of bacteremic pneumonia among US adults. Am J Public Health 2010; 100:1904–11. 20724687 10.2105/AJPH.2009.181313 PMC2936986

[10] Esper AM, Moss M, Lewis CA, et al The role of infection and comorbidity: factors that influence disparities in sepsis. Crit Care Med 2006; 34:2576–82. 16915108 10.1097/01.CCM.0000239114.50519.0E PMC3926300

[11] Mayr FB, Yende S, Linde-Zwirble WT, et al Infection rate and acute organ dysfunction risk as explanations for racial differences in severe sepsis. JAMA 2010; 303:2495–503. 20571016 10.1001/jama.2010.851 PMC3910506

[12] McBean M, Rajamani S Increasing rates of hospitalization due to septicemia in the US elderly population, 1986-1997. J Infect Dis 2001; 183:596–603. 11170985 10.1086/318526

[13] Richardus JH, Kunst AE Black-white differences in infectious disease mortality in the United States. Am J Public Health 2001; 91:1251–3. 11499113 10.2105/ajph.91.8.1251 PMC1446755

[14] Moore JX, Donnelly JP, Griffin R, et al Black-white racial disparities in sepsis: a prospective analysis of the REasons for Geographic And Racial Differences in Stroke (REGARDS) cohort. Crit Care 2015; 19:279. 26159891 10.1186/s13054-015-0992-8 PMC4498511

[15] Oakes JM, Rossi PH The measurement of SES in health research: current practice and steps toward a new approach. Soc Sci Med 2003; 56:769–84. 12560010 10.1016/s0277-9536(02)00073-4

[16] Phelan JC, Link BG, Tehranifar P Social conditions as fundamental causes of health inequalities: theory, evidence, and policy implications. J Health Soc Behav 2010; 51(Suppl):S28–40. 20943581 10.1177/0022146510383498

[17] Kempker JA, Martin GS The changing epidemiology and definitions of sepsis. Clin Chest Med 2016; 37:165–79. 27229635 10.1016/j.ccm.2016.01.002 PMC4884306

[18] National Center for Health Statistics. About the National Health Interview Survey https://www.cdc.gov/nchs/nhis/about_nhis.htm. Accessed 15 March 2017.

[19] National Center for Health Statistics. Office of Analysis and Epidemiology. The National Health Interview Survey (1986–2004) linked mortality files, mortality follow-up through 2006: matching methodology 2009 http://www.cdc.gov/nchs/data/datalinkage/matching_methodology_nhis_final.pdf. Accessed 15 January 2015.

[20] Heron M Deaths: leading causes for 2010. Natl Vital Stat Rep 2013; 20;62(6):1–96. 24364902

[21] VanderWeele TJ Mediation analysis: a practitioner’s guide. Annu Rev Public Health 2016; 37:17–32. 26653405 10.1146/annurev-publhealth-032315-021402

[22] Schonberg MA, Davis RB, McCarthy EP, Marcantonio ER Index to predict 5-year mortality of community-dwelling adults aged 65 and older using data from the National Health Interview Survey. J Gen Intern Med 2009; 24:1115–22. 19649678 10.1007/s11606-009-1073-y PMC2762505

[23] Murphy SL, Xu J, Kochanek KD Deaths: final data for 2015. Natl Vital Stat Rep 2017; 66:1–75. 29235985

