# Supplementary material for: Risk Factors for Septicemia Deaths and Disparities in a Longitudinal US Cohort
Source: Open Forum Infect Dis. 2018 Nov 15;5(12):ofy305. doi: 10.1093/ofid/ofy305 (PMC6290783; doi:10.1093/ofid/ofy305)
Supplement: ofy305_suppl_supplementary_material [file ofy305_suppl_supplementary_material.docx]

**Roles of Health Condition, Health Behavior, and Socioeconomic Factors in Sepsis Deaths - a US Cohort**

**Online Supplement**

**Authors**

Jordan A. Kempker, MD, MSc

Michael R. Kramer, PhD

Lance A. Waller, PhD

Greg S. Martin, MD, MSc

**Contents:**

Contents

[Supplementary Figure 1. Specification of the Study Cohort 2](#_Toc527458771)

[Supplementary Table 1. Hazards of Death by Age Group and Sex, NHIS 1999-2005 (N=206,691) 3](#_Toc527458772)

[Supplementary Table 2. Age Group- and Sex-Adjusted Hazards of Death by Baseline Characteristics, NHIS 1999-2005 (N=206,691) 4](#_Toc527458773)

[Supplementary Table 3. Summary of Comparisons of Age- and Sex-Adjusted Hazards of Different Causes of Death, NHIS 1999-2005 (N=206,691) 9](#_Toc527458774)

[Supplementary Figure 2. Age Group- and Sex-Adjusted Hazard for Death 10](#_Toc527458775)

[Supplementary Figure 3. Age Group- and Sex-Adjusted Health Conditions Hazards for Death 11](#_Toc527458776)

[Supplementary Figure 4. Age Group- and Sex-Adjusted Health Behaviors Hazards for Death 12](#_Toc527458777)

[Supplementary Figure 5. Age Group- and Sex-Adjusted Healthcare Utilization Hazards for Death 13](#_Toc527458778)

[Supplementary Figure 6. Age Group- and Sex-Adjusted Material Capital Hazards for Death 14](#_Toc527458779)

[Supplementary Figure 7. Age Group- and Sex-Adjusted Monetary Capital Hazards for Sepsis Death 15](#_Toc527458780)

[Supplementary Figure 8. Age Group- and Sex-Adjusted Human Capital Hazards for Death 16](#_Toc527458781)

[Supplementary Figure 9. Age Group- and Sex-Adjusted Social Capital Hazards for Death 17](#_Toc527458782)

[Supplementary Table 4. Sex-Adjusted Black-White Hazards Stratified by Age-Group 18](#_Toc527458783)

[Supplementary Table 5. Age-Adjusted Black-White Hazards Stratified by Sex 18](#_Toc527458784)

[Supplementary Table 6. Results of Confounding/Mediation Analyses for the Risk of Septicemia and Other Causes of Death Among Blacks Compared to Whites 19](#_Toc527458785)

[Supplementary Figure 10. Comparisons of the Indirect Mediation on Risk of Death Among Blacks Compared to Whites for Septicemia and Other Causes of Death1 22](#_Toc527458786)

## Supplementary Figure 1. Specification of the Study Cohort

**
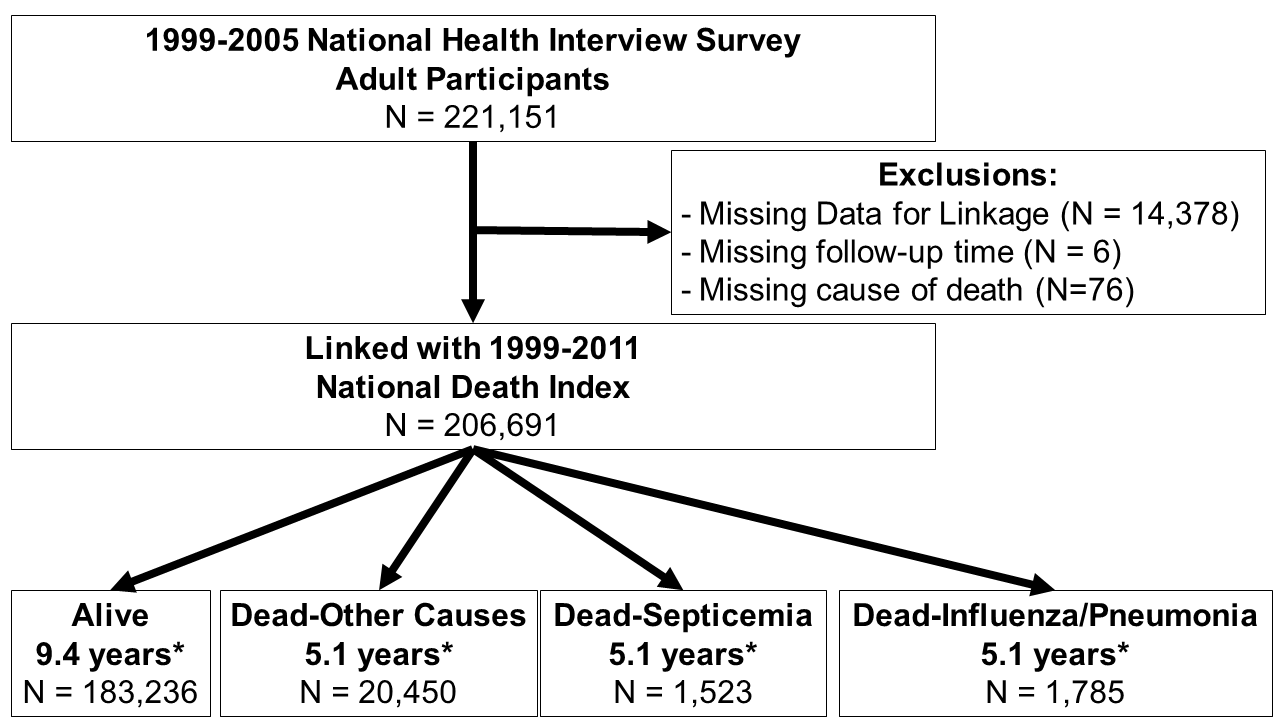
**

*Mean follow-up time

## Supplementary Table 1. Hazards of Death by Age Group and Sex, NHIS 1999-2005 (N=206,691)

| **Variable/Response** | **Influenza & Pneumonia Death**  **HR (95%CI)** | **Septicemia Death**  **HR (95% CI)** | **Non-Sepsis Death**  **HR (95%CI)** |
| --- | --- | --- | --- |
| **Age Group** |  |  |  |
| 17-24 years | Ref | Ref | Ref |
| 25-34 years | 1.48 (0.59-3.7) | 3.81 (1.5-9.69) | 1.41 (1.15-1.73) |
| 35-44 years | 4.99 (2.1-11.84) | 7.24 (2.97-17.62) | 3.26 (2.71-3.92) |
| 45-54 years | 9.14 (3.94-21.22) | 20.82 (8.79-49.34) | 7.69 (6.45-9.16) |
| 55-64 years | 28.26 (12.35-64.66) | 49.54 (21.26-115.44) | 15.88 (13.37-18.87) |
| 65-74 years | 85.28 (38.13-190.77) | 104.67 (44.97-243.58) | 38.01 (32.12-44.97) |
| 75 years and over | 331.87 (147.98-744.27) | 233.12 (100.98-538.15) | 109.57 (92.57-129.69) |
| **Male** | 1.59 (1.44-1.75) | 1.32 (1.17-1.48) | 1.47 (1.42-1.51) |

## Supplementary Table 2. Age Group- and Sex-Adjusted Hazards of Death by Baseline Characteristics, NHIS 1999-2005 (N=206,691)

| **Variable/Response** | **Influenza & Pneumonia**  **HR (95%CI)** | **Septicemia**  **HR (95%CI)** | **Other**  **HR (95%CI)** |
| --- | --- | --- | --- |
| **Human Capital** |  |  |  |
| **Race/Ethnicity** |  |  |  |
| Hispanic | 0.95 (0.77-1.16) | 1.35 (1.13-1.62) | 0.82 (0.78-0.86) |
| Non-Hispanic All other race groups | 0.8 (0.55-1.16) | 1.16 (0.82-1.66) | 0.82 (0.74-0.92) |
| Non-Hispanic Black | 1.14 (0.96-1.36) | 1.92 (1.65-2.23) | 1.32 (1.25-1.38) |
| Non-Hispanic White | Ref | Ref | Ref |
| **Not born in the US** | 0.79 (0.66-0.95) | 0.86 (0.72-1.03) | 0.72 (0.69-0.76) |
| **Not a citizen of the United States** | 0.92 (0.68-1.24) | 1.07 (0.79-1.45) | 0.75 (0.68-0.82) |
| **Language of the interview** |  |  |  |
| English | Ref | Ref | Ref |
| English and Spanish | 0.81 (0.51-1.31) | 1.24 (0.8-1.91) | 0.76 (0.66-0.86) |
| Spanish | 0.98 (0.72-1.34) | 1.2 (0.89-1.62) | 0.81 (0.75-0.88) |
| **Education** |  |  |  |
| Never attended/ kindergarten only | 1.8 (0.75-4.33) | 2.89 (1.19-7.01) | 2.02 (1.56-2.61) |
| Grades 1 - 11 | 3.07 (1.62-5.82) | 2.53 (1.22-5.23) | 2.57 (2.11-3.12) |
| 12th grade, no diploma | 1.46 (0.69-3.1) | 2.52 (1.15-5.57) | 2.22 (1.79-2.76) |
| High School Graduate | 2.09 (1.09-4.01) | 1.78 (0.86-3.67) | 1.93 (1.58-2.35) |
| GED or equivalent | 1.97 (1.01-3.82) | 1.86 (0.87-3.95) | 1.95 (1.59-2.38) |
| Some college, no degree | 2.16 (1.13-4.12) | 1.44 (0.69-3.03) | 1.81 (1.48-2.21) |
| AA degree: technical or vocational | 1.75 (0.88-3.48) | 1.38 (0.63-3) | 1.82 (1.48-2.25) |
| AA degree: academic program | 1.39 (0.59-3.27) | 1.1 (0.46-2.63) | 1.62 (1.29-2.03) |
| Bachelor's degree (BA, AB, BS, BBA) | 1.53 (0.79-2.98) | 1.18 (0.55-2.53) | 1.26 (1.03-1.55) |
| Master's degree (MA, MS, MEng, MEd, MBA) | 1.04 (0.51-2.11) | 0.96 (0.45-2.02) | 1.18 (0.96-1.45) |
| Professional degree (MD, DDS, DVM, JD) | 1.33 (0.59-2.98) | 1.08 (0.4-2.92) | 1.16 (0.92-1.47) |
| Doctoral degree (PhD, EdD) | Ref | Ref | Ref |
|  |  |  |  |
| **Social Capital** |  |  |  |
| **Living with a significant other** | 0.61 (0.54-0.68) | 0.62 (0.55-0.7) | 0.65 (0.63-0.67) |
| **Family type** |  |  |  |
| Multiple adults, ≥ 1 child | 0.64 (0.5-0.84) | 0.6 (0.47-0.77) | 0.63 (0.59-0.67) |
| Multiple adults | 0.73 (0.65-0.81) | 0.78 (0.69-0.88) | 0.73 (0.7-0.76) |
| One adult, ≥ 1 child | 1.39 (0.91-2.13) | 0.82 (0.53-1.27) | 0.81 (0.71-0.91) |
| One adult | Ref | Ref | Ref |
| **Highest education in family** |  |  |  |
| ≤ 8th grade | 2.73 (2.18-3.43) | 2.74 (2.04-3.69) | 2.17 (2.02-2.33) |
| 9-12th grade, no high school diploma | 2.27 (1.82-2.85) | 2.34 (1.74-3.13) | 2.17 (2.02-2.33) |
| High school graduate | 1.92 (1.57-2.35) | 1.95 (1.49-2.55) | 1.75 (1.65-1.86) |
| Some college, no degree | 1.88 (1.51-2.33) | 1.53 (1.16-2.01) | 1.62 (1.52-1.73) |
| AA degree, technical or vocational | 1.53 (1.17-2) | 1.4 (1-1.95) | 1.6 (1.47-1.74) |
| GED recipient | 1.74 (1.34-2.26) | 1.98 (1.47-2.66) | 1.75 (1.62-1.9) |
| AA degree, academic program | 1.54 (0.99-2.39) | 1.54 (1-2.36) | 1.46 (1.31-1.63) |
| Bachelor's degree (BA, BS, AB, BBA) | 1.21 (0.94-1.56) | 1.21 (0.87-1.68) | 1.16 (1.08-1.24) |
| Master's, professional, or doctoral degree | Ref | Ref | Ref |
|  |  |  |  |
| **Material Capital** |  |  |  |
| Individual Earnings, past year |  |  |  |
| $0.00 | 10.36 (4.37-24.56) | 4.15 (2.32-7.43) | 4.29 (3.72-4.95) |
| $01-$4999 | 5.88 (2.29-15.09) | 2.51 (1.28-4.9) | 2.91 (2.48-3.42) |
| $5000-$9999 | 5.53 (2.03-15.06) | 2.85 (1.48-5.5) | 2.47 (2.08-2.93) |
| $10000-$14999 | 4.01 (1.5-10.72) | 2.07 (1.07-4) | 2.26 (1.91-2.67) |
| $15000-$19999 | 3 (1.08-8.3) | 1.58 (0.77-3.23) | 2.17 (1.84-2.56) |
| $20000-$24999 | 4.43 (1.75-11.22) | 0.74 (0.34-1.65) | 1.97 (1.64-2.35) |
| $25000-$34999 | 4.46 (1.76-11.28) | 1.27 (0.66-2.46) | 1.74 (1.49-2.02) |
| $35000-$44999 | 2.08 (0.78-5.56) | 1.55 (0.81-3) | 1.37 (1.15-1.63) |
| $45000-$54999 | 1.18 (0.39-3.6) | 0.94 (0.42-2.08) | 1.34 (1.1-1.63) |
| $55000-$64999 | 1.97 (0.64-6.11) | 0.42 (0.16-1.11) | 0.98 (0.79-1.22) |
| $65000-$74999 | 2.08 (0.5-8.71) | 0.62 (0.17-2.22) | 0.95 (0.72-1.24) |
| $75000 and over | Ref | Ref | Ref |
| **Total combined family income <$20000** |  |  |  |
| **Total combined family income** | 2.66 (1.94-3.63) | 2.8 (2.05-3.81) | 2.49 (2.29-2.71) |
| < $20000 (no detail) | 1.82 (1.37-2.42) | 1.45 (1.09-1.92) | 1.6 (1.48-1.74) |
| ≥$20000 (no detail) | 1.81 (1.6-2.04) | 2.03 (1.79-2.31) | 1.67 (1.62-1.73) |
| 0-$4999 | 3.56 (2.42-5.24) | 2.84 (1.92-4.2) | 2.74 (2.45-3.07) |
| $5000-$9999 | 3.73 (2.77-5.03) | 3.72 (2.82-4.92) | 3.14 (2.9-3.41) |
| $10000-$14999 | 3.17 (2.39-4.2) | 2.32 (1.75-3.08) | 2.73 (2.52-2.96) |
| $15000-$19999 | 2.46 (1.84-3.3) | 2.1 (1.54-2.86) | 2.29 (2.1-2.49) |
| $20000-$24999 | 2.37 (1.77-3.18) | 1.73 (1.27-2.35) | 2.07 (1.91-2.24) |
| $25000-$34999 | 1.88 (1.39-2.54) | 1.6 (1.2-2.15) | 1.96 (1.82-2.12) |
| $35000-$44999 | 1.6 (1.14-2.23) | 1.35 (0.96-1.89) | 1.58 (1.46-1.72) |
| $45000-$54999 | 1.33 (0.93-1.89) | 1.05 (0.72-1.54) | 1.5 (1.36-1.64) |
| $55000-$64999 | 1.15 (0.71-1.86) | 1.03 (0.65-1.63) | 1.24 (1.12-1.37) |
| $65000-$74999 | 1.2 (0.72-2) | 0.78 (0.47-1.31) | 1.3 (1.15-1.46) |
| ≥ $75000 | Ref | Ref | Ref |
| **Poverty index ratio** |  |  |  |
| < 0.50 | 3 (2.1-4.3) | 3.37 (2.41-4.73) | 2.38 (2.14-2.64) |
| .50-.74 | 3.63 (2.64-4.98) | 3.9 (2.98-5.11) | 2.92 (2.67-3.19) |
| .75-.99 | 3.76 (2.86-4.94) | 4.12 (3.21-5.28) | 2.58 (2.38-2.78) |
| 1.00-1.24 | 3.29 (2.52-4.3) | 2.7 (2.05-3.55) | 2.45 (2.26-2.65) |
| 1.25-1.49 | 3.03 (2.35-3.91) | 2.09 (1.54-2.83) | 2.32 (2.15-2.51) |
| 1.50-1.74 | 2.14 (1.63-2.81) | 2.12 (1.55-2.91) | 1.98 (1.82-2.15) |
| 1.75-1.99 | 1.83 (1.33-2.51) | 2.08 (1.53-2.84) | 1.81 (1.66-1.98) |
| 2.00-2.49 | 2.21 (1.74-2.81) | 1.62 (1.24-2.11) | 1.8 (1.68-1.93) |
| 2.50-2.99 | 1.73 (1.29-2.33) | 1.96 (1.48-2.6) | 1.65 (1.52-1.78) |
| 3.00-3.49 | 1.96 (1.45-2.66) | 1.53 (1.11-2.12) | 1.59 (1.46-1.73) |
| 3.50-3.99 | 1.49 (1.07-2.07) | 1.35 (0.95-1.92) | 1.38 (1.26-1.5) |
| 4.00-4.49 | 1.33 (0.88-2.02) | 1.29 (0.86-1.95) | 1.3 (1.18-1.44) |
| 4.50-4.99 | 1.02 (0.64-1.61) | 0.79 (0.5-1.26) | 1.15 (1.03-1.29) |
| ≥ 5.00 | Ref | Ref | Ref |
| **Home ownership** |  |  |  |
| Other arrangement | 1.88 (1.46-2.41) | 1.37 (0.99-1.9) | 1.61 (1.48-1.75) |
| Rented | 1.49 (1.31-1.68) | 1.7 (1.5-1.91) | 1.53 (1.47-1.58) |
| Owned or being bought | Ref | Ref | Ref |
| **No telephone number in house** | 1.49 (1.01-2.19) | 1.65 (1.18-2.3) | 2.02 (1.84-2.21) |
| **No health insurance, currently** | 1.23 (0.93-1.63) | 1.03 (0.8-1.32) | 1.31 (1.23-1.4) |
| **No health insurance any time, past year** | 0.71 (0.38-1.31) | 1.19 (0.76-1.89) | 1.27 (1.13-1.43) |
| **Delayed care in past year bc of worry of cost** | 1.49 (1.21-1.82) | 1.38 (1.15-1.65) | 1.36 (1.28-1.44) |
| **Foregone healthcare in past year bc of cost** | 1.77 (1.42-2.19) | 1.71 (1.36-2.14) | 1.6 (1.5-1.7) |
| **Not filled a prescription bc couldn’t afford it, past year** | 1.75 (1.45-2.11) | 1.79 (1.46-2.2) | 1.63 (1.54-1.73) |
| **How much your family spend on healthcare, past year** |  |  |  |
| $0 | Ref | Ref | Ref |
| < $500 | 0.82 (0.69-0.97) | 0.7 (0.6-0.83) | 0.71 (0.67-0.74) |
| $500-$1,999 | 0.81 (0.68-0.98) | 0.64 (0.54-0.78) | 0.7 (0.66-0.74) |
| $2,000-$2,999 | 0.88 (0.69-1.13) | 0.66 (0.51-0.84) | 0.75 (0.7-0.8) |
| $3,000-$4,999 | 0.78 (0.59-1.02) | 0.74 (0.56-0.99) | 0.81 (0.75-0.88) |
| ≥ $5,000 | 0.7 (0.52-0.95) | 0.81 (0.61-1.07) | 0.91 (0.84-0.99) |
|  |  |  |  |
| **Health Condition** |  |  |  |
| **Past Medical History** |  |  |  |
| Angina, ever | 1.45 (1.23-1.71) | 1.75 (1.41-2.17) | 1.49 (1.41-1.58) |
| Asthma, ever | 1.35 (1.14-1.61) | 1.32 (1.11-1.57) | 1.38 (1.31-1.45) |
| Cancer, past year | 1.22 (1.07-1.38) | 1.3 (1.13-1.5) | 1.43 (1.38-1.49) |
| Chronic bronchitis, past year | 1.89 (1.59-2.24) | 1.55 (1.26-1.9) | 1.67 (1.58-1.76) |
| Diabetes mellitus, past year | 1.46 (1.28-1.67) | 2.32 (2.02-2.66) | 1.76 (1.69-1.82) |
| Emphysema, ever | 2.82 (2.33-3.41) | 2.14 (1.73-2.66) | 2.66 (2.51-2.82) |
| Hay Fever, past year | 1.06 (0.88-1.29) | 0.88 (0.71-1.08) | 0.87 (0.82-0.92) |
| Heart condition, ever | 1.54 (1.35-1.75) | 1.55 (1.36-1.78) | 1.49 (1.43-1.54) |
| Hypertension, ever | 1.2 (1.08-1.34) | 1.78 (1.58-2.01) | 1.34 (1.3-1.38) |
| Limb pain | 1.22 (1.1-1.35) | 1.21 (1.08-1.35) | 1.16 (1.12-1.19) |
| Liver condition, past year | 2.44 (1.74-3.44) | 2.91 (2.12-4) | 2.53 (2.28-2.8) |
| Myocardial infarction, ever | 1.58 (1.37-1.82) | 1.74 (1.48-2.05) | 1.85 (1.77-1.94) |
| Neck pain, 3 months | 1.19 (1.02-1.38) | 1.28 (1.12-1.47) | 1.17 (1.13-1.22) |
| Severe headaches/migraines, 3 months | 1.28 (1.06-1.54) | 1.29 (1.09-1.54) | 1.18 (1.13-1.24) |
| Sinusitis, past year | 1.13 (0.98-1.31) | 0.95 (0.81-1.12) | 1.01 (0.97-1.06) |
| Ulcer, ever | 1.34 (1.19-1.52) | 1.41 (1.21-1.65) | 1.3 (1.24-1.36) |
| Stroke, ever | 1.91 (1.64-2.21) | 2.12 (1.77-2.55) | 2.07 (1.98-2.17) |
| Weak or failing kidneys | 1.91 (1.49-2.44) | 3.98 (3.28-4.82) | 2.67 (2.48-2.87) |
| **Body mass index, kg/m^2^** |  |  |  |
| <18.5 | 1.7 (1.43-2.02) | 1.71 (1.4-2.1) | 1.36 (1.28-1.46) |
| 18.5-24.9 | Ref | Ref | Ref |
| 25-29.9 | 0.66 (0.59-0.75) | 0.86 (0.75-0.99) | 0.8 (0.77-0.82) |
| 30-34.9 | 0.73 (0.62-0.86) | 1.14 (0.97-1.34) | 0.86 (0.83-0.9) |
| ≥ 35 | 1.1 (0.9-1.34) | 1.8 (1.46-2.22) | 1.25 (1.18-1.33) |
| **Any limitation, all conditions** | 2.87 (2.55-3.23) | 3.2 (2.85-3.6) | 2.59 (2.51-2.66) |
| **Any functional limitation, all conditions** | 2.16 (1.9-2.46) | 2.28 (2.01-2.58) | 1.97 (1.9-2.04) |
| **Need help with ADLs** | 4.34 (3.74-5.03) | 4.75 (4.02-5.61) | 3.37 (3.16-3.59) |
| **Need help with instrumental ADLs** | 3.57 (3.14-4.06) | 3.19 (2.78-3.66) | 2.88 (2.75-3.01) |
| **Health problem requires special equipment** | 2.72 (2.39-3.09) | 3.1 (2.72-3.52) | 2.5 (2.41-2.6) |
| **Unable to work due to health problem** | 2.82 (2.5-3.18) | 3.16 (2.8-3.58) | 2.65 (2.56-2.75) |
| **Self-reported general health** |  |  |  |
| Poor | 5.18 (4.11-6.55) | 7.93 (6.15-10.22) | 5.31 (4.98-5.67) |
| Fair | 3 (2.43-3.71) | 4.73 (3.78-5.93) | 2.95 (2.79-3.11) |
| Good | 1.8 (1.47-2.2) | 2.75 (2.21-3.41) | 1.87 (1.78-1.97) |
| Very good | 1.22 (0.98-1.51) | 1.5 (1.18-1.91) | 1.34 (1.27-1.41) |
| Excellent | Ref | Ref | Ref |
| **Health compared to year ago** |  |  |  |
| Better | 1.12 (0.96-1.31) | 1.3 (1.12-1.52) | 1.19 (1.13-1.24) |
| About the same | Ref | Ref | Ref |
| Worse | 2 (1.75-2.29) | 1.93 (1.68-2.21) | 1.98 (1.9-2.06) |
| **Days health kept in bed, past year** |  |  |  |
| None | Ref | Ref | Ref |
| 1-7 days | 1.12 (0.96-1.3) | 1.04 (0.88-1.22) | 1.09 (1.05-1.14) |
| >7 days | 2.19 (1.9-2.52) | 2.44 (2.12-2.82) | 2.16 (2.08-2.25) |
|  |  |  |  |
| **Health Behaviors** |  |  |  |
| **Tobacco use status** |  |  |  |
| Never smoker | Ref | Ref | Ref |
| Former smoker | 1.35 (1.21-1.5) | 1.28 (1.13-1.45) | 1.29 (1.25-1.34) |
| Current some day smoker | 1.92 (1.43-2.58) | 1.8 (1.31-2.46) | 1.95 (1.79-2.13) |
| Current every day smoker | 2.07 (1.8-2.39) | 2.02 (1.74-2.36) | 2.41 (2.31-2.51) |
| **Cigarettes per day all participants** |  |  |  |
| None | Ref | Ref | Ref |
| 1/2 ppd | 1.7 (1.4-2.05) | 1.57 (1.27-1.93) | 1.83 (1.74-1.93) |
| 1/2-1 ppd | 1.64 (1.34-2) | 1.71 (1.42-2.06) | 2.12 (2.01-2.23) |
| > 1 ppd | 2.46 (1.85-3.28) | 2.36 (1.83-3.04) | 2.85 (2.65-3.06) |
| **Alcohol Use** |  |  |  |
| Lifetime abstainer [<12 drinks, lifetime] | Ref | Ref | Ref |
| Former drinker [No drinks, past year] | 1.12 (0.98-1.27) | 1.22 (1.06-1.39) | 1.25 (1.2-1.3) |
| Current drinker [≥1 drinks, past year] | 0.63 (0.55-0.72) | 0.71 (0.62-0.81) | 0.8 (0.77-0.83) |
| **Alcohol use, days per week^[[1]](#footnote-1)^** |  |  |  |
| None | Ref | Ref | Ref |
| 1-2 days/week | 0.62 (0.52-0.75) | 0.65 (0.53-0.79) | 0.74 (0.71-0.78) |
| 3-5 days/week | 0.58 (0.44-0.77) | 0.48 (0.35-0.66) | 0.73 (0.68-0.78) |
| 6-7 days/week | 0.69 (0.56-0.85) | 0.77 (0.6-0.98) | 0.92 (0.87-0.98) |
| **Chance of getting AIDS virus** |  |  |  |
| None | Ref | Ref | Ref |
| Low | 0.92 (0.78-1.09) | 0.86 (0.73-1.02) | 0.98 (0.93-1.02) |
| Medium | 1.33 (0.78-2.28) | 0.87 (0.37-2.06) | 1.1 (0.93-1.31) |
| High/Already have AIDS virus | 0.67 (0.25-1.82) | 1.07 (0.44-2.58) | 1.53 (1.26-1.87) |
| **Light or moderate activity** |  |  |  |
| None | Ref | Ref | Ref |
| 1-3 times/week | 0.59 (0.5-0.69) | 0.54 (0.46-0.63) | 0.58 (0.56-0.6) |
| 4-7 times/week | 0.59 (0.52-0.67) | 0.55 (0.46-0.65) | 0.6 (0.58-0.62) |
| >7 times/week | 0.7 (0.49-1) | 0.58 (0.4-0.85) | 0.74 (0.66-0.82) |
| **Muscle strengthening activity** |  |  |  |
| None | Ref | Ref | Ref |
| 1-3 times/week | 0.48 (0.36-0.62) | 0.62 (0.48-0.8) | 0.59 (0.55-0.63) |
| 4-7 times/week | 0.8 (0.62-1.04) | 0.69 (0.53-0.88) | 0.68 (0.63-0.73) |
| >7 times/week | 0.78 (0.36-1.66) | 0.93 (0.47-1.86) | 0.95 (0.76-1.17) |
| **Vigorous activity** |  |  |  |
| None | Ref | Ref | Ref |
| 1-3 times/week | 0.38 (0.3-0.48) | 0.42 (0.34-0.52) | 0.51 (0.48-0.54) |
| 4-7 times/week | 0.38 (0.29-0.51) | 0.35 (0.26-0.47) | 0.5 (0.46-0.53) |
| >7 times/week | 0.3 (0.11-0.8) | 0.84 (0.37-1.92) | 0.61 (0.48-0.77) |
|  |  |  |  |
| **Healthcare Utilization** |  |  |  |
| **Has usual place for healthcare** | 1 (0.77-1.31) | 1.13 (0.89-1.42) | 0.97 (0.92-1.03) |
| **Has consulted with primary care provider, past year** | 1.14 (1-1.31) | 1.15 (1-1.32) | 1.13 (1.08-1.17) |
| **Number of office visits, past year** |  |  |  |
| None | Ref | Ref | Ref |
| 1 | 0.76 (0.58-1) | 0.92 (0.7-1.2) | 0.86 (0.81-0.92) |
| 2-3 times | 1 (0.81-1.24) | 0.83 (0.67-1.02) | 0.87 (0.82-0.92) |
| >3 times | 1.35 (1.11-1.65) | 1.62 (1.34-1.96) | 1.36 (1.29-1.44) |
| **Seen health care professional, past 2 weeks** | 1.26 (1.13-1.4) | 1.4 (1.24-1.59) | 1.32 (1.28-1.36) |
| **Spoken to healthcare professional, past 2 weeks** | 1.38 (1.18-1.6) | 1.33 (1.1-1.61) | 1.32 (1.26-1.38) |
| **Received healthcare > 10 times, past year** | 1.87 (1.68-2.08) | 2.4 (2.15-2.68) | 1.89 (1.83-1.95) |
| **Been overnight in hospital, past year** | 1.98 (1.75-2.25) | 2.32 (2.05-2.62) | 1.98 (1.91-2.06) |
| **Overnight hospital stays, past year** |  |  |  |
| None | Ref | Ref | Ref |
| 1 | 1.82 (1.57-2.1) | 1.91 (1.64-2.22) | 1.66 (1.59-1.73) |
| > 1 | 2.43 (2.01-2.94) | 3.4 (2.83-4.08) | 2.87 (2.71-3.03) |
| **Number of ER visits past year** |  |  |  |
| None | Ref | Ref | Ref |
| 1 | 1.47 (1.28-1.68) | 1.54 (1.32-1.79) | 1.45 (1.39-1.52) |
| 2-3 times | 2.14 (1.8-2.54) | 2.26 (1.86-2.75) | 2 (1.89-2.12) |
| >3 times | 2.42 (1.79-3.28) | 3.56 (2.79-4.55) | 2.88 (2.64-3.13) |
| **Received pneumonia vaccine in lifetime** | 1.14 (1.02-1.28) | 1.17 (1.01-1.34) | 1.18 (1.14-1.23) |
| **Received influenza vaccine, past year** | 1.14 (1.01-1.28) | 1.11 (0.98-1.26) | 1.07 (1.03-1.1) |

## Supplementary Table 3. Summary of Comparisons of Age- and Sex-Adjusted Hazards of Different Causes of Death, NHIS 1999-2005 (N=206,691)

| **Septicemia**  **≈**  **Influenza/Pneumonia**  **≈**  **Other Causes** | **Septicemia HR**  **≠**  **Other Causes HR** | **Influenza/Pneumonia HR**  **≠**  **Other Causes HR** |
| --- | --- | --- |
|  |  |  |
| Not born in the US | Race/Ethnicity | Need help with IADLs |
| Not a citizen of the United States | Hypertension | Alcohol Use |
| Language of the interview | Body mass index | Cigarettes per day |
| Education | Self-reported general health | Family type |
| Living with a significant other | Unable to work due to health problem |  |
| Home ownership | Health problem requires special equipment |  |
| No telephone number in house | Any limitation |  |
| No health insurance any time, past year | Alcohol use, days per week |  |
| Delayed care bc of worry of cost, past year | Received healthcare > 10 times, past year |  |
| Foregone healthcare bc of cost, past year |  |  |
| Angina |  |  |
|  | **Septicemia HR and Influenza/Pneumonia HR**  **≠**  **Other Causes HR** | |
| Asthma | **Same Direction** | |
| Cancer | Diabetes mellitus | |
| Chronic bronchitis | Weak or failing kidneys | |
| Emphysema |  | |
| Hay Fever | **Different Directions** | |
| Heart condition | Need help with ADLs | |
| Limb pain | Poverty index ratio | |
| Liver condition |  |  |
| Myocardial infarction |  |  |
| Neck pain |  |  |
| Severe headaches/migraines |  |  |
| Sinusitis |  |  |
| Ulcer |  |  |
| Stroke |  |  |
| Any functional limitation |  |  |
| Health compared to year ago |  |  |
| Days health kept in bed, past year |  |  |
| Light or moderate activity |  |  |
| Muscle strengthening activity |  |  |
| Vigorous activity |  |  |
| Has usual place for healthcare |  |  |
| Number of office visits, past year |  |  |
| Overnight hospital stays, past year |  |  |
| Number of ER visits past year |  |  |
| Received pneumonia vaccine in lifetime |  |  |
| Received influenza vaccine, past year |  |  |

## Supplementary Figure 2. Age Group- and Sex-Adjusted Hazard for Death

**
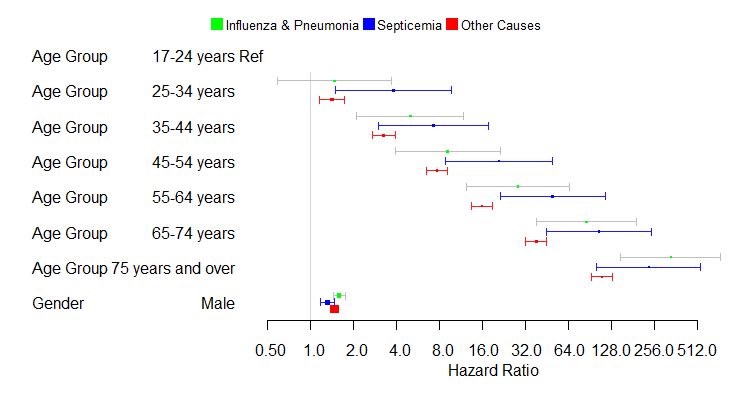
**

## Supplementary Figure 3. Age Group- and Sex-Adjusted Health Conditions Hazards for Death


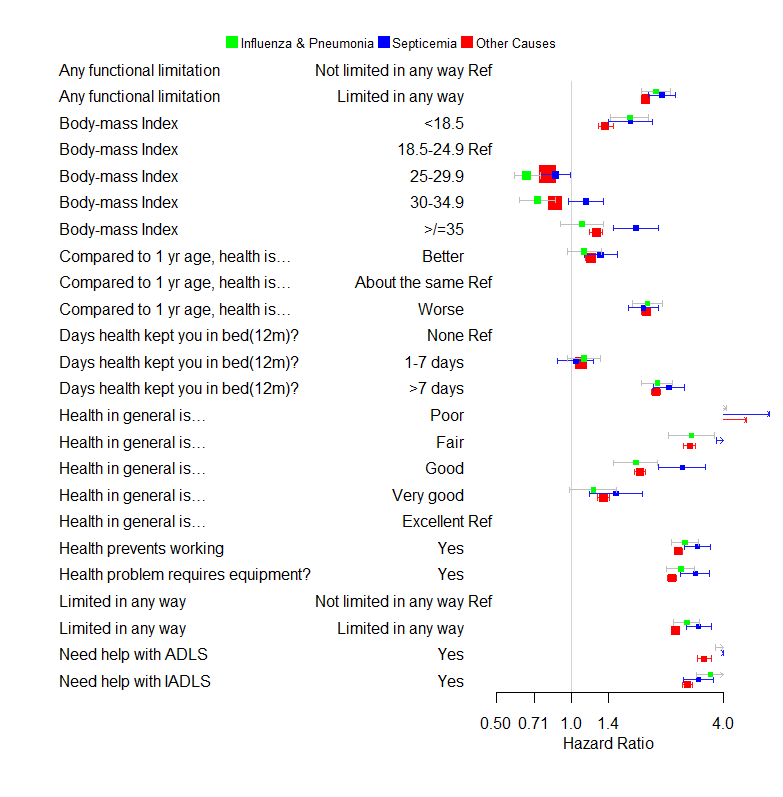


## Supplementary Figure 4. Age Group- and Sex-Adjusted Health Behaviors Hazards for Death


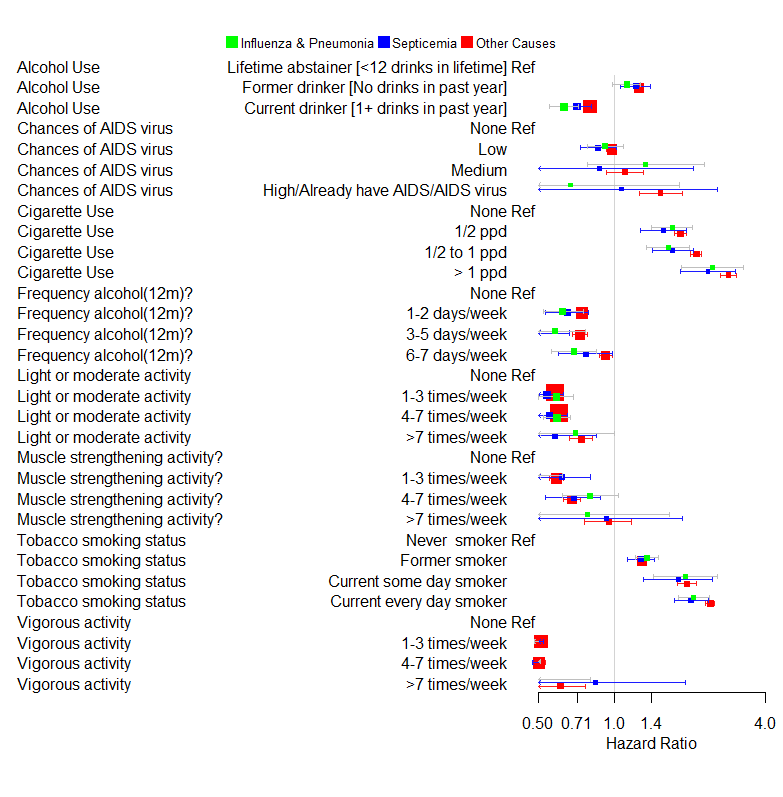


## Supplementary Figure 5. Age Group- and Sex-Adjusted Healthcare Utilization Hazards for Death


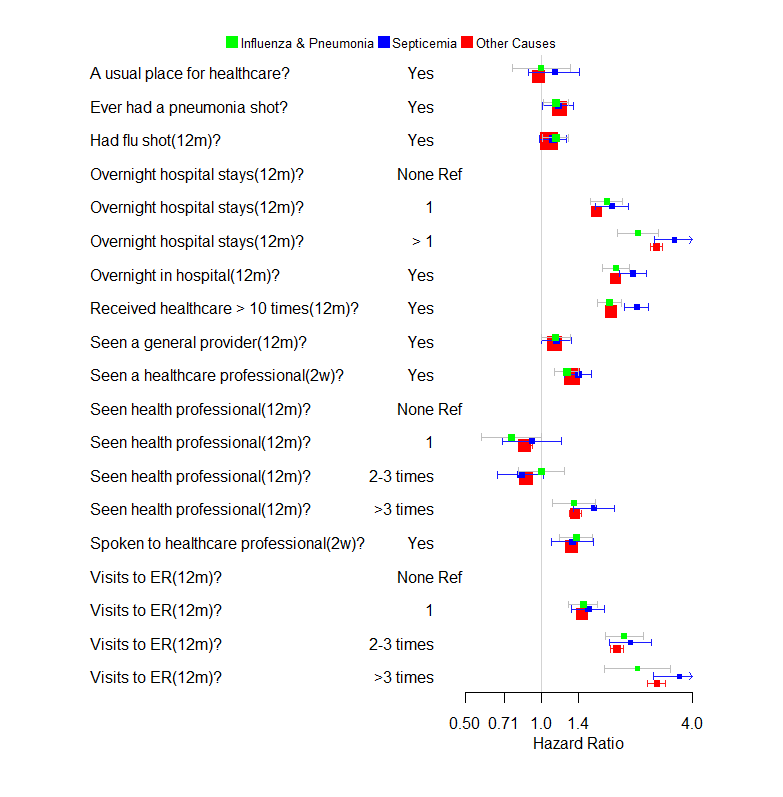


## Supplementary Figure 6. Age Group- and Sex-Adjusted Material Capital Hazards for Death


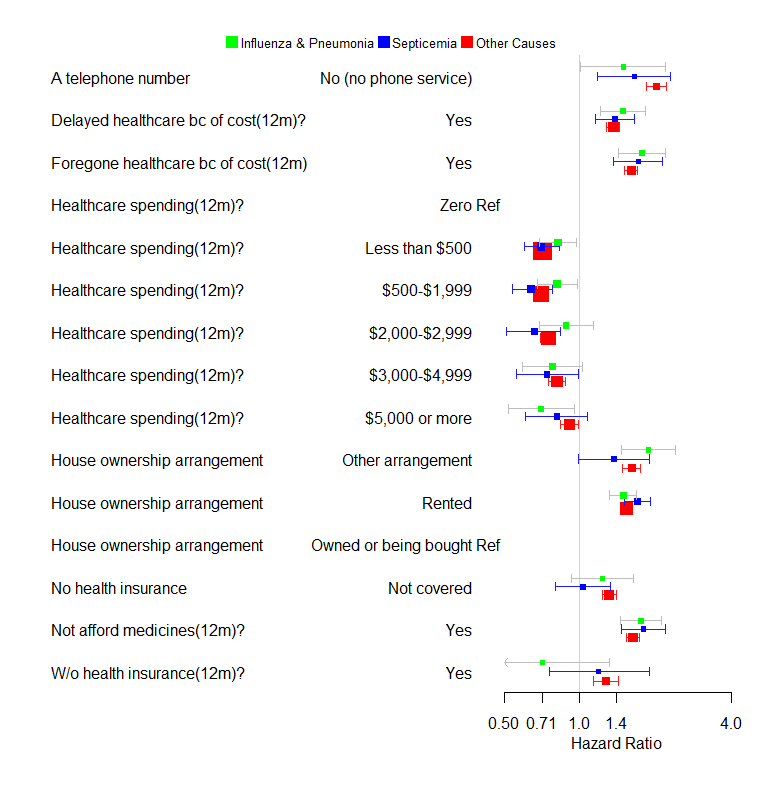


## Supplementary Figure 7. Age Group- and Sex-Adjusted Monetary Capital Hazards for Sepsis Death


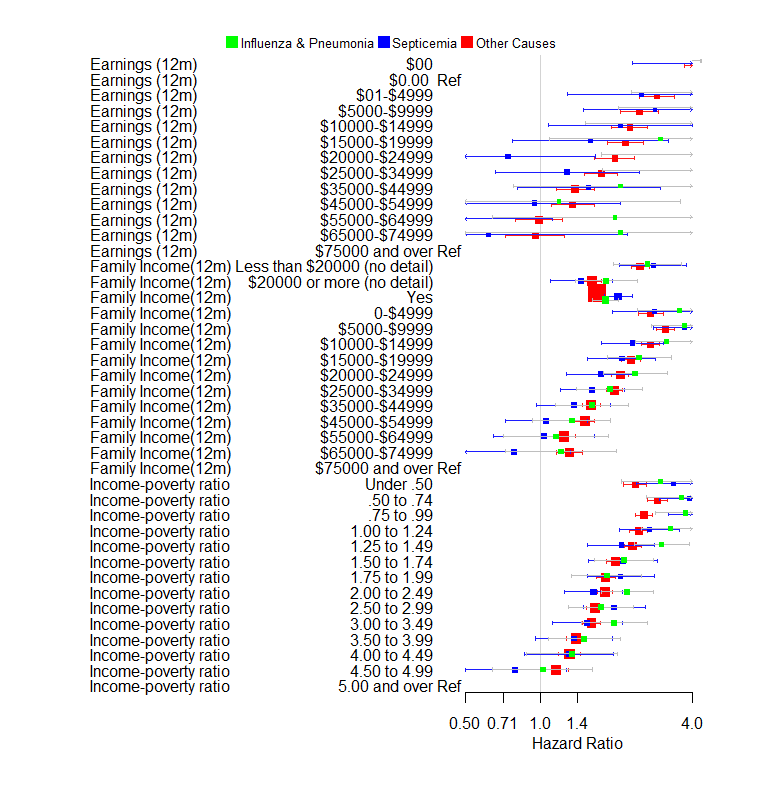


## Supplementary Figure 8. Age Group- and Sex-Adjusted Human Capital Hazards for Death


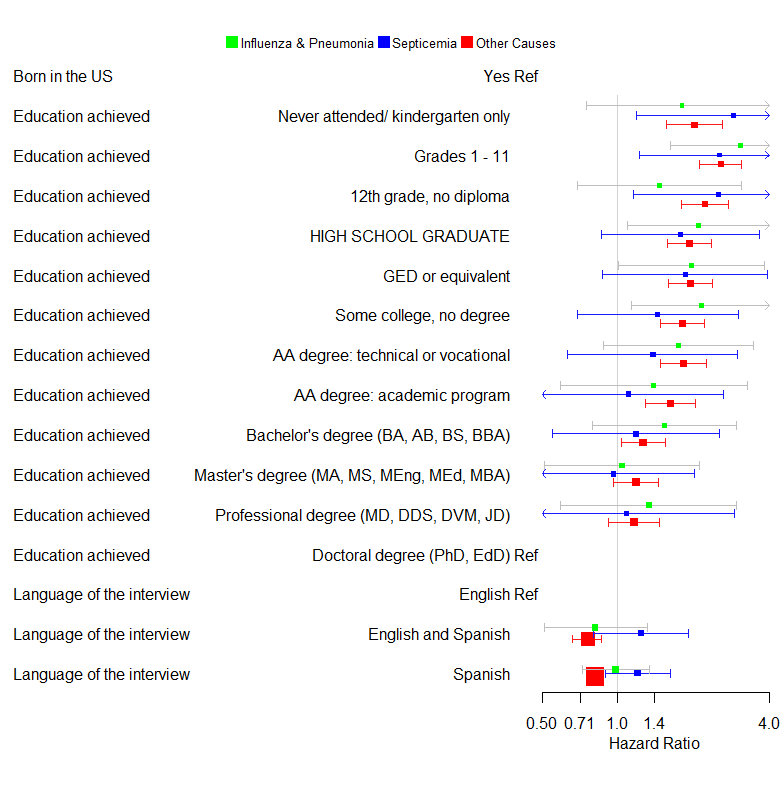


## Supplementary Figure 9. Age Group- and Sex-Adjusted Social Capital Hazards for Death


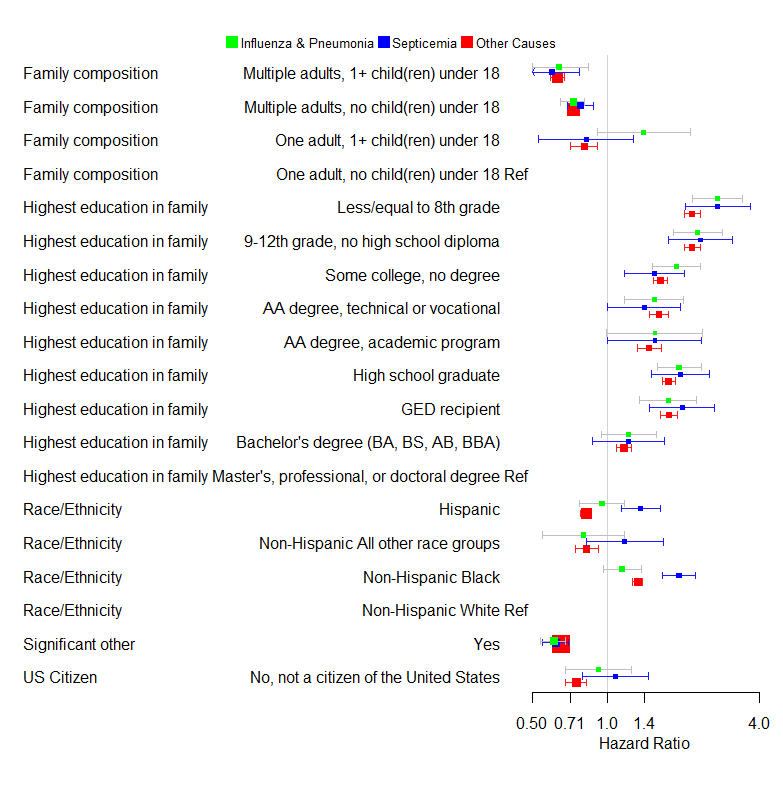


## Supplementary Table 4. Sex-Adjusted Black-White Hazards Stratified by Age-Group

| **Age Strata, years** | **Sex-Adjusted Black-White Hazard Ratio For Sepsis Death Within Each Age Strata**  **(95% CI)** | **P** | **Sex-Adjusted Black-White Hazard Ratio For Influenza-Pneumonia Death Within Each Age Strata**  **(95% CI)** | **P** |
| --- | --- | --- | --- | --- |
| **17-24** | 4.1 (0.5-31.4) | 0.1763 | 0 (0-0) | <.0001 |
| **25-34** | 2.5 (1.0-6.3) | 0.0502 | 1.3 (0.2-7.0) | 0.7719 |
| **35-44** | 1.8 (0.9-3.4) | 0.0959 | 1.5 (0.7-3.1) | 0.2589 |
| **45-54** | 2.3 (1.6-3.5) | <.0001 | 2.0 (1.2-3.3) | 0.0077 |
| **55-64** | 1.8 (1.2-2.7) | 0.0064 | 1.8 (1.1-2.9) | 0.0112 |
| **65-74** | 1.9 (1.4-2.5) | <.0001 | 1.1 (0.8-1.5) | 0.5002 |
| **≥75** | 1.9 (1.5-2.3) | <.0001 | 0.9 (0.7-1.2) | 0.5786 |

## Supplementary Table 5. Age-Adjusted Black-White Hazards Stratified by Sex

| **Sex Strata** | **Age-Adjusted Black-White Hazard Ratio For Sepsis Death Within Each Sex Strata**  **(95% CI)** | **P** | **Age-Adjusted Black-White Hazard Ratio For Influenza/Pneumonia Death Within Each Sex Strata**  **(95% CI)** | **P** |
| --- | --- | --- | --- | --- |
| **Female** | 1.8 (1.5-2.3) | <.0001 | 1.1 (0.9-1.4) | 0.2372 |
| **Male** | 2.0 (1.6-2.5) | <.0001 | 1.1 (0.9-1.5) | 0.3199 |

## Supplementary Table 6. Results of Confounding/Mediation Analyses for the Risk of Septicemia and Other Causes of Death Among Blacks Compared to Whites

|  | **Septicemia Deaths**  **Total Age- & Sex-Adjusted**  **Race Effect = 0.65** | | | **Other Causes of Death**  **Total Age- & Sex-Adjusted**  **Race Effect = 0.28** | | |
| --- | --- | --- | --- | --- | --- | --- |
|  | **Direct Effect of Race after covariate adjustment** | **Indirect Race Effect Mediated through covariate^1^** | **Proportion of Total Race Effect Mediated through covariate^2^** | **Direct Effect of Race after covariate adjustment** | **Indirect Race Effect Mediated through covariate^1^** | **Proportion of Total Race Effect Mediated through covariate^2^** |
| **Human Capital** |  |  |  |  |  |  |
| Education achieved | 0.53 | 0.12 | 0.18 | 0.16 | 0.11 | 0.41 |
| Language of the interview | 0.64 | 0.01 | 0.01 | 0.28 | 0 | 0 |
| Born in the US | 0.65 | 0 | -0.01 | 0.28 | 0 | -0.01 |
|  |  |  |  |  |  |  |
| **Material Capital** |  |  |  |  |  |  |
| Income-poverty ratio | 0.4 | 0.25 | 0.38 | 0.1 | 0.18 | 0.64 |
| Family Income(12m) | 0.47 | 0.18 | 0.28 | 0.17 | 0.1 | 0.38 |
| Family Income(12m) | 0.47 | 0.18 | 0.28 | 0.12 | 0.16 | 0.57 |
| Family Income(12m) | 0.53 | 0.12 | 0.19 | 0.17 | 0.1 | 0.38 |
| Family Income(12m) | 0.53 | 0.12 | 0.19 | 0.12 | 0.16 | 0.57 |
| House ownership arrangement | 0.54 | 0.11 | 0.16 | 0.18 | 0.1 | 0.36 |
| Earnings (12m) | 0.61 | 0.04 | 0.07 | 0.22 | 0.06 | 0.21 |
| Not afford medicines (12m)? | 0.63 | 0.02 | 0.03 | 0.26 | 0.02 | 0.07 |
| Foregone healthcare bc of cost(12m) | 0.63 | 0.02 | 0.03 | 0.26 | 0.02 | 0.07 |
| Healthcare spending (12m)? | 0.65 | 0 | 0.01 | 0.27 | 0.01 | 0.03 |
| Delayed healthcare bc of cost (12m)? | 0.64 | 0.01 | 0.01 | 0.27 | 0.01 | 0.02 |
| W/o health insurance (12m)? | 0.66 | -0.01 | -0.01 | 0.27 | 0.01 | 0.02 |
| No health insurance | 0.66 | -0.01 | -0.01 | 0.27 | 0.01 | 0.04 |
| A telephone number | 0.67 | -0.02 | -0.02 | 0.25 | 0.03 | 0.1 |
|  |  |  |  |  |  |  |
| **Social Capital** |  |  |  |  |  |  |
| Highest education in family | 0.55 | 0.1 | 0.16 | 0.17 | 0.11 | 0.38 |
| Significant other | 0.56 | 0.09 | 0.14 | 0.2 | 0.08 | 0.29 |
| Family composition | 0.64 | 0.01 | 0.02 | 0.25 | 0.03 | 0.1 |
| US Citizen | 0.65 | 0 | 0 | 0.28 | 0 | -0.01 |
|  |  |  |  |  |  |  |
| **Health Behavior** |  |  |  |  |  |  |
| Light or moderate activity | 0.56 | 0.09 | 0.14 | 0.2 | 0.08 | 0.28 |
| Alcohol Use | 0.6 | 0.05 | 0.08 | 0.21 | 0.06 | 0.23 |
| Vigorous activity | 0.6 | 0.05 | 0.08 | 0.23 | 0.05 | 0.18 |
| Frequency alcohol (12m)? | 0.63 | 0.02 | 0.03 | 0.25 | 0.03 | 0.11 |
| Muscle strengthening activity? | 0.64 | 0.01 | 0.01 | 0.26 | 0.01 | 0.05 |
| Tobacco smoking status | 0.66 | -0.01 | -0.02 | 0.27 | 0 | 0.01 |
| Cigarette Use | 0.67 | -0.02 | -0.03 | 0.28 | 0 | 0 |
| Chances of AIDS virus | 0.68 | -0.03 | -0.05 | 0.29 | -0.01 | -0.04 |
|  |  |  |  |  |  |  |
| **Health Condition** |  |  |  |  |  |  |
| Health in general is… | 0.41 | 0.24 | 0.37 | 0.08 | 0.2 | 0.71 |
| Health prevents working | 0.51 | 0.14 | 0.21 | 0.16 | 0.11 | 0.42 |
| Limited in any way | 0.54 | 0.11 | 0.17 | 0.19 | 0.09 | 0.32 |
| Health problem requires equipment? | 0.55 | 0.1 | 0.16 | 0.2 | 0.08 | 0.29 |
| Need help with IADLS | 0.56 | 0.09 | 0.14 | 0.19 | 0.09 | 0.32 |
| Need help with ADLS | 0.57 | 0.08 | 0.13 | 0.22 | 0.06 | 0.22 |
| Body-mass Index | 0.61 | 0.04 | 0.06 | 0.27 | 0.01 | 0.02 |
| Days health kept you in bed (12m)? | 0.63 | 0.02 | 0.02 | 0.27 | 0.01 | 0.03 |
| Compared to 1 yr age, health is… | 0.64 | 0.01 | 0.01 | 0.27 | 0.01 | 0.03 |
| Any functional limitation | 0.64 | 0.01 | 0.01 | 0.26 | 0.01 | 0.04 |
|  |  |  |  |  |  |  |
| **Medical Comorbidity** |  |  |  |  |  |  |
| Diabetes mellitus | 0.56 | 0.09 | 0.14 | 0.22 | 0.06 | 0.21 |
| Hypertension | 0.57 | 0.08 | 0.12 | 0.23 | 0.04 | 0.15 |
| Weak or failing kidneys | 0.61 | 0.04 | 0.06 | 0.26 | 0.02 | 0.07 |
| Stroke | 0.63 | 0.02 | 0.03 | 0.26 | 0.02 | 0.06 |
| Asthma | 0.65 | 0 | 0.01 | 0.28 | 0 | 0 |
| Hay Fever | 0.65 | 0 | 0 | 0.27 | 0 | 0.01 |
| Severe headache/migraine (3m)? | 0.65 | 0 | 0 | 0.27 | 0 | 0.01 |
| Chronic bronchitis | 0.65 | 0 | 0 | 0.28 | 0 | -0.01 |
| Liver condition | 0.65 | 0 | 0 | 0.27 | 0 | 0.01 |
| Sinusitis(12m) | 0.65 | 0 | 0 | 0.28 | 0 | 0 |
| Neck pain | 0.66 | 0 | -0.01 | 0.28 | 0 | -0.02 |
| Stomach or intestinal ulcer | 0.66 | -0.01 | -0.01 | 0.28 | 0 | -0.02 |
| Angina | 0.67 | -0.01 | -0.02 | 0.29 | -0.01 | -0.04 |
| Myocardial infarction | 0.66 | -0.01 | -0.02 | 0.29 | -0.01 | -0.05 |
| Limb pain | 0.66 | -0.01 | -0.02 | 0.28 | 0 | -0.01 |
| Emphysema | 0.67 | -0.02 | -0.03 | 0.29 | -0.02 | -0.06 |
| Heart condition | 0.67 | -0.02 | -0.03 | 0.3 | -0.02 | -0.07 |
| Cancer | 0.69 | -0.03 | -0.05 | 0.31 | -0.04 | -0.14 |
| **Healthcare Utilization** |  |  |  |  |  |  |
| Visits to ER (12m)? | 0.59 | 0.06 | 0.09 | 0.23 | 0.05 | 0.17 |
| Overnight hospital stays (12m)? | 0.62 | 0.03 | 0.04 | 0.26 | 0.02 | 0.07 |
| Received healthcare > 10 times (12m)? | 0.63 | 0.02 | 0.03 | 0.26 | 0.01 | 0.05 |
| Overnight in hospital (12m)? | 0.64 | 0.01 | 0.02 | 0.27 | 0.01 | 0.03 |
| Seen a healthcare professional (2w)? | 0.65 | 0 | 0 | 0.28 | 0 | 0 |
| A usual place for healthcare? | 0.65 | 0 | 0 | 0.28 | 0 | 0 |
| Seen health professional (12m)? | 0.66 | -0.01 | -0.01 | 0.28 | -0.01 | -0.02 |
| Seen a general provider (12m)? | 0.66 | 0 | -0.01 | 0.28 | 0 | -0.01 |
| Spoken to healthcare professional (2w)? | 0.66 | -0.01 | -0.01 | 0.28 | -0.01 | -0.03 |
| Had flu shot (12m)? | 0.68 | -0.03 | -0.05 | 0.29 | -0.02 | -0.06 |
| Ever had a pneumonia shot? | 0.69 | -0.04 | -0.06 | 0.3 | -0.02 | -0.09 |

^1^A positive value in this column is indicative of a covariate reducing the parameter estimate (and thus the hazard ratio) of the risk of death in Blacks compared to Whites upon its addition to the age- and sex-adjusted model. This can be indicative of confounding or mediation, depending on the underlying causal structure not revealed in this analysis. A negative value in this column is indicative of increasing the parameter estimate (and thus hazard ratio) of the risk of death in Blacks compared to Whites upon its addition to the age- and sex-adjusted model.

^2^ A positive value in this column is indicative of a covariate reducing the parameter estimate (and thus the hazard ratio) of the risk of death in Blacks compared to Whites upon its addition to the age- and sex-adjusted model. This can be indicative of confounding or mediation, depending on the underlying causal structure not revealed in this analysis. A negative value in this column is indicative of increasing the parameter estimate (and thus hazard ratio) of the risk of death in Blacks compared to Whites upon its addition to the age- and sex-adjusted model. This column represents a proportion, and therefore interpretation of comparisons across causes of death must take into account that the age- and sex-adjusted parameter estimate (denominator of the proportion) for death of Blacks compared to Whites is smaller for ‘Other Causes of Death’ than it is for ‘Septicemia Death’.

## Supplementary Figure 10. Comparisons of the Indirect Mediation on Risk of Death Among Blacks Compared to Whites for Septicemia and Other Causes of Death1


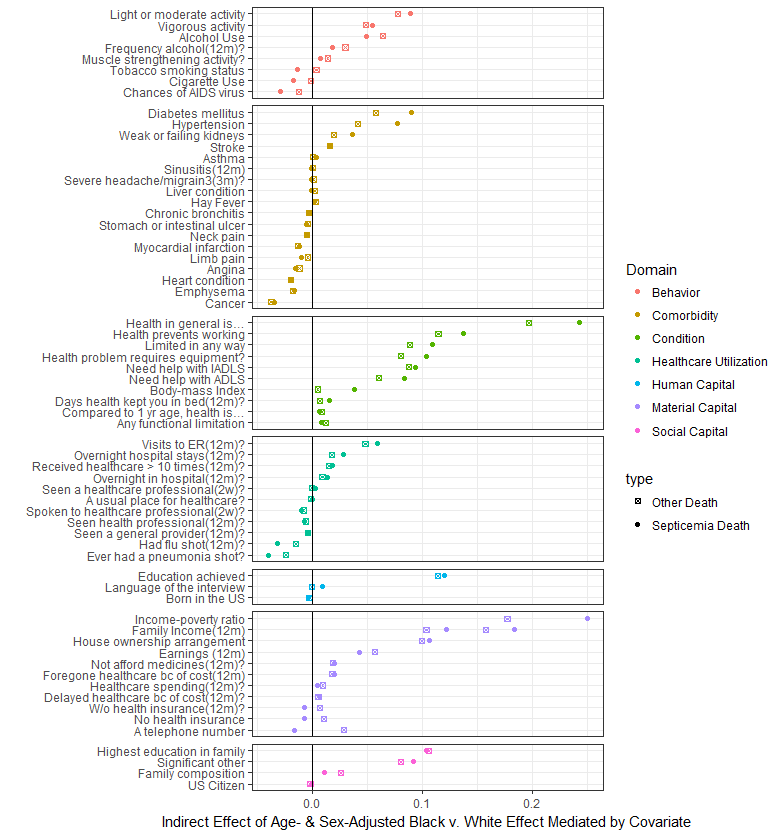


^1^The *indirect effects* measures for this graph are calculated by subtraction from the Black-White parameter estimate in a Cox Proportional hazards adjusting for age and sex of the Black-White parameter estimate in a model including age, sex, and the additional variable indicated on the y-axis of the graph. This is performed separately for septicemia and other causes of death to calculate cause-of-death specific indirect effects for each variable. The indirect effect is conceptualized as the absolute amount of the age- and sex-adjusted Black-White disparity accounted for by the indicated variable.

1. Lifetime abstainers and those who hadn’t drank in past year recoded as zero. [↑](#footnote-ref-1)
